# Molecular Targets and Biological Activities of Carvone: An Evidence-Graded Review

**DOI:** 10.3390/molecules31173106

**Published:** 2026-09-04

**Authors:** Zdeněk Dvořák, Jiří Hrubý

**Affiliations:** Department of Cell Biology and Genetics, Faculty of Science, Palacký University, Šlechtitelů 27, 783 71 Olomouc, Czech Republic; jiri.hruby01@upol.cz

**Keywords:** carvone, monoterpene ketone, aryl hydrocarbon receptor, enantiomers, molecular targets, biological activities, Nrf2, GABA-A receptor

## Abstract

Carvone is a naturally occurring monoterpene and the principal volatile constituent of spearmint (*Mentha spicata*) and caraway (*Carum carvi*), plants long used as carminative, digestive, antimicrobial, and flavoring agents. The compound exists as two enantiomers—*S*-(+)-carvone (predominantly associated with caraway) and *R*-(−)-carvone (predominantly associated with spearmint)—widely used in food, fragrance, and herbal preparations. Although carvone is a naturally occurring compound, the material used in experimental studies may be synthetically produced, isolated from plant essential oils, or used as a racemic or stereochemically enriched preparation. This review synthesizes evidence from 108 literature sources, complemented by PubChem (1535 records, accessed on 14 July 2026) and ChEMBL (136 records, accessed on 14 July 2026) bioassay data, to compile carvone’s molecular targets and biological activities and align the pharmacological evidence with traditional uses. We identified 45 molecular targets and 99 documented biological activities across 38 classes, spanning evidence tiers from comprehensively characterized (Tier A/B) to screening-level observations (Tier C/D), each assigned a four-tier evidence grade (A/B/C/D). Tier A targets comprise the aryl hydrocarbon receptor (AhR) and the GABA-A receptor. Negative results are reported to prevent publication bias, including NCI-60 inactivity across 55 cancer cell lines and a comprehensive counterscreen of steroid/nuclear receptors, human protein kinases, phosphatases, and HSP90—all inactive. The aryl hydrocarbon receptor emerges as the most robustly characterized target by the evidence-tiering criteria: both enantiomers act as noncompetitive allosteric antagonists, validated in vivo in mouse skin. This designation reflects the breadth and quality of evidence for AhR antagonism specifically, not a claim that AhR mediates all of carvone’s traditional or pharmacological effects. By mapping mechanistic evidence onto carvone’s traditional carminative, antimicrobial, and topical applications, this work provides a plausible mechanistic basis for several of the empirical uses documented in the ethnopharmacological literature.

## 1. Introduction

**Figure 1:**
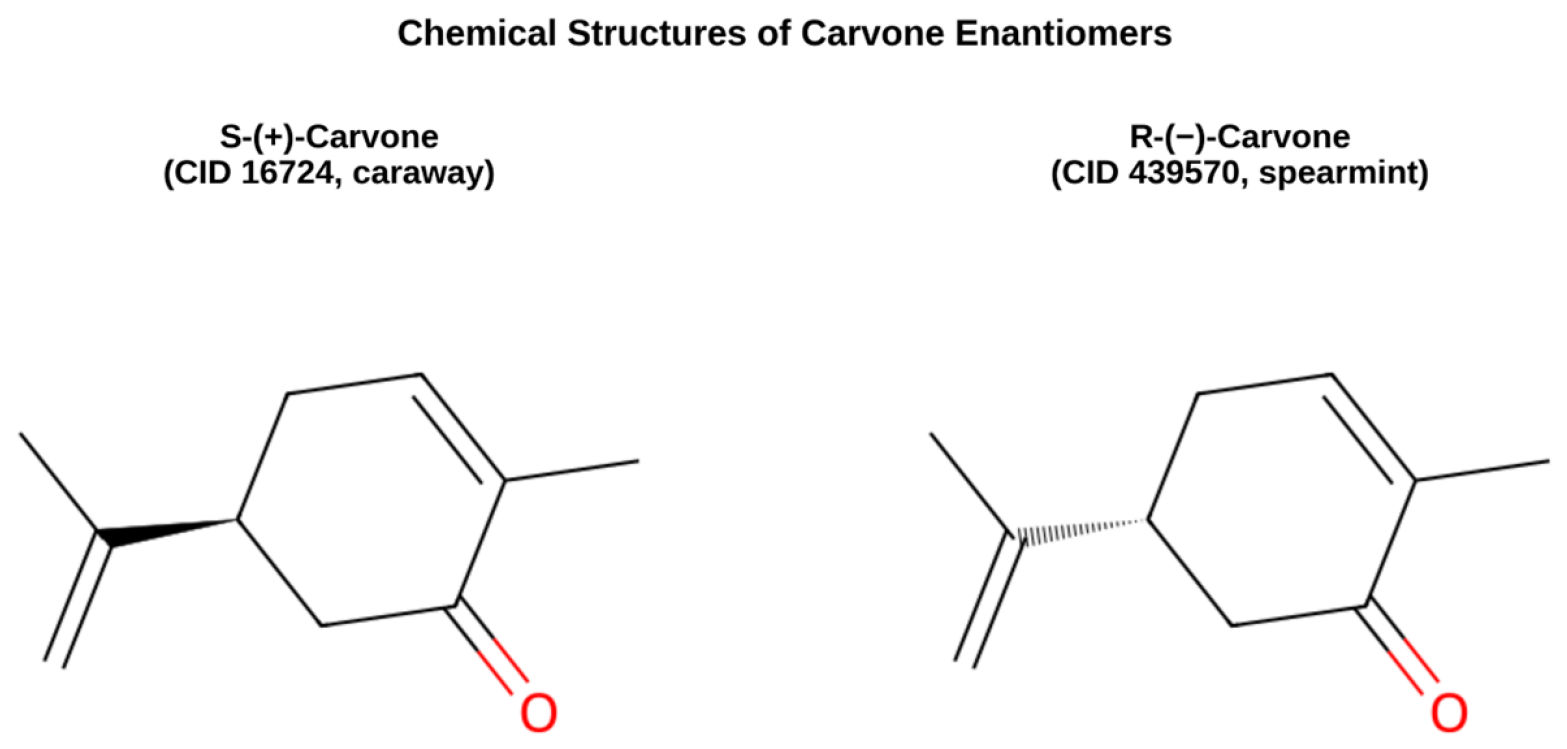
Chemical structures of carvone enantiomers: *S*-(+)-carvone (**left**, CID 16724, predominantly associated with caraway) and *R*-(−)-carvone (**right**, CID 439570, predominantly associated with spearmint). The stereocenter at C2 is indicated by wedge/dash bond notation.

Carvone is a monocyclic monoterpene ketone (MW 150.22) that constitutes the primary volatile component of spearmint oil (60–70%) and caraway seed oil (50–60%). The compound features a stereocenter at C2, giving rise to two enantiomers (Figure 1): *S*-(+)-carvone (PubChem CID 16724), predominantly associated with caraway, and *R*-(−)-carvone (PubChem CID 439570), predominantly associated with spearmint. While these enantiomer–plant associations are predominant, they are not absolute; the stereochemical composition can vary with cultivar, growing conditions, and extraction method. An unspecified racemic form (CID 7439) is also encountered in industrial contexts. Although carvone is a naturally occurring compound, the material used in experimental studies may be synthetically produced, isolated from plant essential oils, or used as a racemic or stereochemically enriched preparation. The source and stereochemical composition of carvone used in each cited study are noted where specified in the original reports. Both enantiomers are approved for food use by the FDA and are widely employed as flavoring agents, fragrances, and in traditional herbal medicine. Spearmint and caraway have documented traditional uses as carminative, digestive, antimicrobial, and food-preservative agents across multiple cultures, with caraway fruits recognized in the European Union herbal monograph for symptomatic relief of digestive disorders (bloating and flatulence) and spearmint traditionally used for cold, cough, asthma, fever, and digestive problems [1,2]. Carvone-rich essential oils are also used in modern carminative preparations, topical formulations, and transdermal patches as skin permeabilizers.

The biological activity of carvone has been investigated across an unusually broad range of model systems—from molecular binding assays to in vivo disease models—yielding a fragmented but extensive literature. Several factors motivate a comprehensive synthesis at this time. First, carvone is a widely consumed monoterpene in the human diet [3,4], yet its pharmacological targets have not been systematically catalogued. Second, recent studies have identified the aryl hydrocarbon receptor (AhR) as a high-affinity target with potential therapeutic relevance in skin protection and immunomodulation [5,6,7]. Third, the compound’s enantiomeric forms are often studied independently, making it difficult to assess whether observed activities are enantiomer-specific or shared. Fourth, carvone’s pesticidal and antimicrobial properties have attracted interest as natural alternatives to synthetic agents [8,9,10,11,12,13]. Fifth, from an ethnopharmacological perspective, there is a need to systematically connect carvone’s empirically documented traditional uses—carminative, digestive, antimicrobial, and topical—with their molecular pharmacological basis, the core question that this review addresses. While Pina et al. (2022) systematically reviewed carvone’s pharmacological activities [4], the present review extends this work in four respects: (i) it integrates PubChem and ChEMBL bioassay data alongside the literature, providing a more comprehensive target inventory; (ii) it introduces a four-tier evidence-grading framework (A/B/C/D) to stratify findings by robustness; (iii) it catalogues molecular targets separately from biological activities, enabling mechanistic analysis; and (iv) it explicitly maps the graded mechanistic evidence onto carvone’s traditional ethnopharmacological uses.

This review compiles evidence from three complementary data sources: (i) an exhaustive PubMed literature search; (ii) PubChem bioassay data comprising 1535 assay records; and (iii) ChEMBL activity data comprising 136 records. The resulting dataset encompasses 108 references, 45 distinct molecular targets, and 99 documented biological activities across 38 classes, spanning evidence tiers from comprehensively characterized (Tier A/B) to screening-level observations (Tier C/D). The detailed retrieval and data-processing workflow is described in Materials and Methods (Section 8).

### Traditional and Ethnopharmacological Uses

Carvone is the dominant volatile constituent of several aromatic plants with well-documented traditional medicinal use. Spearmint (*Mentha spicata L.*, Lamiaceae) essential oil contains *R*-(−)-carvone and has a long history of use for digestive complaints, cold, cough, asthma, fever, and as a flavoring agent; a recent systematic review in this journal confirms that the pharmacological properties of *M. spicata* support its traditional uses, with antimicrobial, antioxidant, anti-inflammatory, and hepatoprotective activities consistently reported [2]. Caraway (*Carum carvi L*., Apiaceae) seed oil contains *S*-(+)-carvone and is recognized in the European Union herbal monograph (EMA/HMPC) for symptomatic relief of digestive disorders, including bloating and flatulence; its carminative and digestive effects are considered the superior traditional actions, and caraway oil (often combined with peppermint oil or menthol in enteric-coated preparations) has been documented in clinical studies for functional dyspepsia [1]. Dill (*Anethum graveolens*) seed essential oil is another carvone-rich traditional remedy with documented antimicrobial and carminative use [14]. Carvone-chemotype *Lippia alba* (Verbenaceae) is used traditionally in Latin America for digestive and metabolic complaints [15]. Beyond medicinal use, carvone’s antimicrobial and antioxidant properties have supported its role as a natural food preservative and flavoring agent, and carvone-rich essential oils are employed in fragrance and cosmetic formulations [1]. In modern product contexts, carvone is encountered in over-the-counter carminative preparations, mint extracts, essential-oil blends, and transdermal patches where it serves as a skin permeabilizer [3]. The two enantiomers map onto the two principal traditional botanical sources: *S*-(+)-carvone characterizes caraway, while *R*-(-)-carvone characterizes spearmint—a correspondence that frames the enantiomer-selective pharmacology discussed later. A recent systematic review of carvone’s pharmacological activities confirms antibacterial, antispasmodic, anti-inflammatory, and digestive effects across multiple experimental models, consistent with these traditional applications [4]. The following sections compile the molecular targets and biological activities that now provide a mechanistic basis for these empirical uses. It should be noted that carvone-rich essential oils contain multiple bioactive constituents, and the observed effects of whole-plant preparations cannot be attributed solely to carvone.

## 2. Molecular Targets

Carvone interacts with 45 distinct molecular targets, which can be grouped into eight functional categories. The targets span nuclear receptors and transcription factors, metabolic and signaling enzymes, ion channels, chemosensory receptors, GPCRs and other receptors, kinase cascades, protein interactions and other screening hits, and pathogen proteins. Each target is assigned an evidence tier (A/B/C/D; see Section 8.7) reflecting the robustness of supporting evidence: Tier A (two targets, AhR and GABA-A) denotes comprehensive multi-assay characterization with in vivo validation; Tier B (23 targets) denotes solid dose–response or in vivo evidence; Tier C (11 targets) denotes qHTS screening flags without independent confirmation; and Tier D (nine targets) denotes artifacts, in silico-only predictions, or unverifiable records. This section describes the principal targets within each category, with evidence tiers noted to guide interpretation.

### 2.1. Nuclear Receptors and Transcription Factors

The aryl hydrocarbon receptor (AhR) is the most extensively characterized carvone target. Both enantiomers act as noncompetitive, insurmountable allosteric antagonists of the human AhR, with K_i_ values of 11–48 µM in AZ-AhR reporter cells. Binding occurs at the bHLH domain, preventing AhR-ARNT heterodimerization and nuclear translocation. In vivo, topical *S*-carvone inhibited benzo[*a*]pyrene-induced Cyp1a1 mRNA expression in mouse skin and protected against UV-induced sunburn cell formation [5]. Two recent studies from the Dvořák group extended this work: one screened 100 monoterpenoids for AhR antagonist activity, identifying structural determinants (carbonyl position, molecular planarity, C3/C5-alkylation) and identifying 3-methyl-*S*-carvone as a lead pan-antagonist [7]; the other demonstrated that methylcarvones exert immunomodulatory effects through AhR antagonism [6] (Table 1).

Carvone also modulates several other transcription factors. NF-κB is inhibited through two distinct mechanisms: *S*-carvone activates SIRT1, leading to p65 deacetylation and transcriptional repression [16], while *R*-carvone induces Nrf2 nuclear translocation and HO-1 expression, sequestering CBP/p300 from NF-κB [17]. The Nrf2/ARE pathway is thus a central node in carvone’s antioxidant and anti-inflammatory actions. Several additional nuclear receptor targets have been flagged in PubChem qHTS screening, but a critical data-quality review shows these require cautious interpretation. Thyroid hormone receptor beta (TRβ, PubChem AID 588547) and the ARE/Nrf2 reporter (PubChem AID 651741) returned “inconclusive” outcomes with artifact phenotypes (cytotoxic and fluorescent, respectively), invalidating their curve-fit potency values; these should not be considered validated targets. Retinoid-related orphan receptor gamma (RORγ, PubChem AID 651802) and constitutive androstane receptor (CAR/NR1I3, PubChem AID 1794836) are inconclusive qHTS hits of low confidence. Estrogen receptor alpha (ERα, PubChem AID 743075) could not be independently verified via the PubChem API; however, Ondrová et al. [5] directly tested *S*-carvone against ERα by radioligand binding and found <50% radioligand displacement (inactive), providing orthogonal evidence that carvone does not bind ERα. None of these qHTS hits have been confirmed by orthogonal dose–response assays as carvone agonists/antagonists, and none should be interpreted as established carvone targets. In addition to ERα, Ondrová et al. [5] performed a radioligand binding counterscreen of *S*-carvone against the androgen receptor (AR), ERα, ERβ, progesterone receptor (PR), glucocorticoid receptor (GR), retinoid X receptor alpha (RXRα), and peroxisome proliferator-activated receptor gamma (PPARγ)—with all being inactive. The Tier D designation applies specifically to a PubChem ARE/Nrf2 reporter qHTS assay that was flagged as an artifact due to assay interference (fluorescent phenotype contradicting the assay design). This database artifact designation does not affect the Nrf2 pathway activation documented in peer-reviewed literature by Sousa et al. [17], which remains valid as Tier B evidence (dose–response quantification in RAW264.7 macrophages with HO-1 induction > 35-fold).

**Table 1:** Molecular targets of carvone: Nuclear receptors and transcription factors. Sub-rows represent distinct assays, measures, or studies. PubChem and ChEMBL assay IDs are cited in the Ref column and are not included in the reference list. Evidence tier: A = comprehensive characterization; B = solid single method; C = screening flag; D = weak/unverified.

Target	Activity Type	Measure/Value	Enantiomer	Species/Model	Ref	Tier
Antioxidant response element (ARE)/Nrf2	Agonist (qHTS, INCONCLUSIVE)	Curve-fit AC_50_ (fluorescent artifact)	*S*	Human (luciferase reporter)	PubChem AID 651741	D
Aryl hydrocarbon receptor (AhR)	Allosteric antagonist	K_i_ (vs. TCDD-activated AhR)	*S*, *R*	Human (AZ-AhR cells)	[5]	A
Aryl hydrocarbon receptor (AhR)	Allosteric antagonist	Binding (microscale thermophoresis)	*S*, *R*	Human (recombinant protein)	[5]	A
Aryl hydrocarbon receptor (AhR)	Antagonist (in vivo proof of concept)	inhibition of BaP-induced Cyp1a1 mRNA	*S*	Mouse (skin, in vivo)	[5]	A
Constitutive androstane receptor (CAR)	Antagonist (qHTS, INCONCLUSIVE)	Curve-fit AC_50_ (inconclusive)	*S*	Human (luciferase reporter)	PubChem AID 1794836	C
Estrogen receptor alpha (ERα)	Agonist (qHTS, unverified—data not retrievable)	Reported potency (unverified)	*S*	Human (luciferase reporter)	PubChem AID 743075	D
MyD88-dependent signaling (AKI)	Inhibitor (pathway)	Protein expression (WB): (significant)	*S*	Mouse (LPS-induced AKI)	[18]	B
NF-κB (transcriptional activity via JNK1/Nrf2)	Inhibitor (target gene expression)	Significant decrease in IκB-α resynthesis	*R*	Mouse (RAW264.7 macrophages)	[17]	B
NF-κB/caspase-3 pathway	Inhibitor (NF-κB) + anti-apoptotic (caspase-3)	Protein expression (WB): (significant)	*S*	Mouse (glycerol-induced rhabdomyolysis AKI)	[19]	B
Nuclear factor erythroid 2-related factor 2 (Nrf2/NFE2L2)	Activator (nuclear translocation)	increase in nuclear Nrf2	*R*	Mouse (RAW264.7 macrophages)	[17]	B
Retinoid-related orphan receptor gamma (RORγ)	Antagonist (qHTS, INCONCLUSIVE)	Curve-fit AC_50_ (low confidence)	*S*	Human (luciferase reporter)	PubChem AID 651802	C
SREBP1/TLR4 pathway (metabolic/ovarian)	Modulator (pathway)	Protein/gene expression: Active (dose-dependent)	Unspecified (from *M. spicata*)	Mouse (PCOS model)	[20]	B
Sirtuin-1 (SIRT1)	Direct activator	increased SIRT1 activity	*S*	Human (recombinant SIRT1)	[16]	B
TLR4/NF-κB/Nrf2/HO-1 pathway (lung)	Inhibitor (TLR4/NF-κB) + Activator (Nrf2/HO-1)	Protein expression (WB): (significant)	*S*	Rat (LPS-induced acute lung injury)	[21]	B
TLR4/NLRP3 inflammasome pathway	Inhibitor (pathway)	mRNA/protein expression (WB, PCR): (significant)	*S*	Rat (cerebral ischemia/reperfusion model)	[22]	B
Thyroid hormone receptor beta (TRβ)	Antagonist (qHTS, INCONCLUSIVE)	Curve-fit AC_50_ (cytotoxic curve)	*S*	Human (luciferase reporter)	PubChem AID 588547	D

### 2.2. Enzymes

Acetylcholinesterase (AChE) is inhibited by *S*-carvone with a K_i_ of 300 µM (PubChem AID 1098346; literature-deposited via ChEMBL from a study in Industrial Crops and Products), a finding relevant to both pesticidal and potential neuroprotective applications. PubChem flags this value with a “data validity: outside typical range” comment, so it should be interpreted with caution. Carvone inhibits cytochrome P450 enzymes: *S*-carvone inhibits CYP2D6 (18 µM, PubChem AID 1671196)—the only PubChem qHTS result for carvone classified as genuinely “active” (activity score 81, efficacy 95%)—and both enantiomers show inconclusive inhibition of CYP1A2 (~64 µM, PubChem AID 1671199), suggesting drug–drug interaction potential that warrants confirmatory study. A nematode CYP450 (Bx-cyp29A3) responds to carvone exposure [23] (Table 2).

Other enzyme targets identified through qHTS screening include aldehyde dehydrogenase 1A1 (ALDH1A1, ChEMBL CHEMBL3577) and matrix metalloproteinase-9 (MMP9, in silico docking only). A ChEMBL data-quality audit revealed that the ALDH1A1 qHTS returned one “Inconclusive” and one “Not Active” result for *S*-carvone, meaning carvone did not reliably inhibit ALDH1A1. These qHTS enzyme hits should not be considered validated targets and require confirmatory dose–response assays. Two further negative findings from the [5] counterscreen delineate carvone’s selectivity among non-AhR signaling proteins: S-carvone did not displace geldanamycin from the chaperone HSP90, and it did not inhibit the non-receptor tyrosine phosphatases PTPN11/SHP2 or PTPN6/SHP1.

### 2.3. Ion Channels, Neurotransmitter and Chemosensory Receptors

The GABA-A receptor is a key neurological target. Both carvone enantiomers act as negative allosteric modulators. In a flunitrazepam binding assay, carvone shifted the GABA EC_50_ from a basal 5 µM to 90 µM (*R*-carvone, 16-fold shift) and 15 µM (*S*-carvone, 3-fold shift), with reduced maximal response [25]. *R*-carvone is thus the more potent GABA-A negative modulator. This GABA-A modulation partially explains carvone’s insecticidal activity, as GABA-A is a major insecticide target. *S*-carvone also acts as an anesthetic at clinically relevant concentrations, positively modulating GABA-A while inhibiting NMDA receptors and Nav1.2 voltage-gated sodium channels [26,27]. Additional ion channel targets include TRPV1 (*R*-carvone agonist, calcium influx) [28], TRP channels in the spinal dorsal horn (modulating glutamate release) [29], and L-type calcium channels (antiarrhythmic effects in cardiac tissue) [30] (Table 3).

Carvone enantiomers differentially activate the human odorant receptor OR1A1, with *S*-carvone more potent than *R*-carvone in a head-to-head comparison [31]. *R*-carvone activates the murine olfactory receptor Olfr1259 in pancreatic beta-cells, enhancing glucose-stimulated insulin secretion via cAMP/Ca^2+^ signaling [32]. *R*-carvone also inhibits bitter taste receptors TAS2R31/TAS2R43 [33], suggesting potential applications in taste masking. These findings demonstrate that chemosensory receptors have metabolic signaling roles beyond olfaction.

**Table 3:** Molecular targets of carvone: Ion channels and neurotransmitter and chemosensory receptors. Sub-rows represent distinct assays, measures, or studies. PubChem and ChEMBL assay IDs are cited in the Ref column and are not included in the reference list. Evidence tier: A = comprehensive characterization; B = solid single method; C = screening flag; D = weak/unverified.

Target	Activity Type	Measure/Value	Enantiomer	Species/Model	Ref	Tier
GABA-A receptor	Negative allosteric modulator	shift of GABA EC_50_ for flunitrazepam binding	*S*, *R*	Rat (primary cortical neurons)	[25]	A
GABA-A receptor (anesthetic modulation)	Positive modulator	Xenopus oocyte, 2-electrode voltage clamp): Active	*S*	Rat (Xenopus oocytes, recombinant)	[26]	B
L-type calcium channel (cardiac, antiarrhythmic)	Inhibitor (ICa,L)	(patch clamp)	*R*	Rat (isolated hearts, ventricular myocyte)	[30]	B
NMDA receptor (anesthetic modulation)	Negative modulator	Xenopus oocyte, 2-electrode voltage clamp): Active	*S*	Rat (Xenopus oocytes, recombinant)	[26]	B
Nav1.2 (voltage-gated sodium channel)	Inhibitor	Xenopus oocyte, 2-electrode voltage clamp): Active	*S*	Rat (Xenopus oocytes, recombinant)	[26]	B
TRP channels (spinal dorsal horn)	Activator (enhances *L*-glutamate release)	Electrophysiology (spontaneous EPSC frequency): Active	*R*, *S*	Rat (spinal cord substantia gelatinosa)	[29]	B
TRPV1 channel (transient receptor potential vanilloid 1)	Agonist	calcium influx, DRG neurons): Active	*R*	Rat (DRG neurons)/Human (HEK293)	[28]	B
Voltage-gated sodium channels	Blocker (putative, inferred from nerve excitability data)	reduction in compound action	*R*	Rat (isolated sciatic nerve)	[27]	B
Bitter taste receptor TAS2R31/TAS2R43	Inhibitor (antagonist)	Ca^2+^ response; inhibits bitter perception	*R*	Human (HEK293-TAS2R cells)	[34]	B
Odorant receptor OR1A1	Agonist (enantioselective)	receptor activation, cAMP;: Different *R* > *S*	*S*, *R*	Human (Hana3A cells, heterologous)	[31]	B
Olfactory receptor Olfr1259	Agonist (promotes insulin secretion)	Increased Ca^2+^ and cAMP; enhanced GSIS	*R*	Mouse (β-TC6 pancreatic cells)	[32]	B
Adrenergic beta-2 receptor (ADRB2)	Agonist (qHTS, INCONCLUSIVE)	Curve-fit AC_50_ (from inconclusive curve)	*S*	Human (luciferase reporter)	PubChem AID 1963593	D

Table 3 footnote—GABA-A receptor Tier A justification: The GABA-A receptor is designated Tier A based on convergent evidence from four independent assay systems: (i) displacement of [^3^H]-flunitrazepam binding in rat cortical neurons, demonstrating competitive interaction at the benzodiazepine site [25]; (ii) electrophysiological confirmation in Xenopus oocytes expressing recombinant GABA-A receptors, showing negative allosteric modulation of GABA-evoked currents [26]; (iii) in vivo anesthetic activity in sheep, with carvone producing reversible anesthesia at doses consistent with the in vitro potency [26,35,36]; and (iv) antinociceptive effects in mice, providing independent in vivo behavioral confirmation [27]. The convergence of binding, electrophysiological, and two independent in vivo behavioral endpoints across different species satisfies the Tier A criterion of multi-assay characterization with in vivo validation. JNK1, by contrast, is designated Tier B because the evidence is limited to cell-based phosphorylation assays in RAW264.7 macrophages [16,17] without in vivo target-specific measurement.

### 2.4. Kinase and Signaling Pathways

Carvone modulates multiple kinase cascades. *R*-carvone inhibits JNK1 phosphorylation in RAW264.7 macrophages [16,17] (Tier B: cell-based evidence without in vivo target validation) and p38 MAPK [37]. The GSK-3β/PKC-α pathway is targeted in cardiac tissue, where carvone attenuates fibrosis and inflammation [38]. In breast cancer, *S*-carvone inhibits the FAK/Src pathway, reducing cell adhesion, migration, and invasion [39]. Hepatic fibrosis is mitigated through TGF-β/Smad pathway inhibition [40] (Table 4). Several inflammatory signaling cascades are also modulated: TLR4/NLRP3 inflammasome in cerebral ischemia [22], TLR4/NF-κB/Nrf2/HO-1 in acute lung injury [21], TLR4/MyD88 in acute kidney injury [18], and NF-κB/caspase-3 in rhabdomyolysis-induced AKI [19]. The cAMP/PKA pathway is inhibited in melanogenesis [41]. To place these specific kinase effects in context, Ondrová et al. [5] profiled *S*-carvone (100 µM) against 468 human protein kinases using the KINOMEscan scanMAX active-site-directed competition binding assay (35% of control activity = inhibition threshold); 467 of 468 kinases remained above the cutoff, with only TK2 (a JH2-domain pseudokinase not involved in transcriptional regulation) inhibited to 27% of control. Separately, protein kinase C (PKC) catalytic activity was unaffected in HepG2 lysates up to 1000 µM, ruling out PKC inhibition as the mechanism of carvone’s AhR suppression. These counterscreen results indicate that the JNK1 and p38 MAPK inhibition reported above does not reflect promiscuous kinase inhibition but rather a narrow, high-concentration effect on specific kinases.

Carvone also inhibits quorum sensing (CviR/CviI system), blocking biofilm formation [43,44]. Microtubules/tubulin are targeted by *R*-carvone, exhibiting allelopathic activity [42].

## 3. Biological Activities

The 99 documented biological activities of carvone, spanning evidence tiers from comprehensively characterized (Tier A/B) to screening-level observations (Tier C/D), span 38 classes that can be grouped into 10 therapeutic areas. Each activity is assigned an evidence tier (A/B/C/D; see Section 8.7): Tier A (five activities, all nephroprotective in vivo studies with multiple endpoints and dose–response) denotes comprehensive characterization; Tier B (72 activities) denotes dose–response quantification or in vivo validation; Tier C (21 activities) denotes single-endpoint screening or qualitative activity; and Tier D (two activities) denotes the excluded qHTS artifact and an in silico-only entry. This section synthesizes the evidence by activity class, with evidence tiers noted to distinguish robustly characterized activities from screening-level observations.

### 3.1. Anti-Inflammatory Activity

Carvone exhibits potent anti-inflammatory activity through two mechanistically distinct pathways that converge on NF-κB inhibition. Sousa et al. demonstrated that *S*-carvone activates SIRT1 in RAW264.7 macrophages, leading to p65 deacetylation and reduced production of NO, IL-1β, and IL-6 [16]. In an ongoing study, they showed that *R*-carvone induces Nrf2 nuclear translocation and HO-1 expression, sequestering CBP/p300 from NF-κB, while also inhibiting JNK1 phosphorylation [17]. A head-to-head comparison found *S*-carvone more potent than *R*-carvone for NO inhibition [45]. Additional anti-inflammatory studies include carvone attenuation of LPS-induced acute lung injury via TLR4/NF-κB/Nrf2/HO-1 [21], inhibition of eosinophil airway infiltration [46], and intestinal anti-inflammatory effects against DSS-induced colitis [47] and gastric ulcer [48] (Table 5).

### 3.2. Antimicrobial Activity

Carvone shows broad-spectrum antimicrobial activity. Both enantiomers exhibit synergism when combined with gentamycin [8], colistin [9], and other antibiotics [10].

*S*-carvone demonstrates anticandidal activity [49] and antibiofilm activity against *S. aureus* [11]. Carvone inhibits virulence factors and biofilm in *Serratia marcescens* [50]. A carvone nanoemulgel targeting MRSA has been developed [51], and antifungal activity against *Candida* and *Aspergillus* species has been demonstrated [52,53,54,55]. The primary antimicrobial mechanism involves membrane disruption. Carvone’s lipophilic monoterpene structure intercalates into microbial membranes, increasing permeability and causing leakage of intracellular contents. Synergy with aminoglycosides suggests that membrane disruption enhances drug uptake [8]. Anti-virulence [50] and anti-bacterial activity [43] involve quorum sensing inhibition (Table 6).

Carvone shows antiviral activity against SARS-CoV-2 with an EC_50_ of 86 µM [57]. In silico docking studies confirm carvone interaction with the SARS-CoV-2 spike protein RBD [58]. Carvone also exhibits anti-virulence activity against *S. marcescens* [50] and anti-*Toxoplasma gondii* activity (IC_50_ 16 µg/mL; ChEMBL). The antiviral mechanism may involve AhR antagonism, as AhR has been identified as a pro-viral host factor for coronaviruses.

### 3.3. Cytotoxic and Anticancer Activity

Carvone shows enantiomer- and cell-type-dependent cytotoxicity. *R*-carvone induces p53/caspase-3-mediated apoptosis and inhibits migration in breast cancer cells (MCF-7, MDA-MB-231) [59]. Carvone causes ROS-mediated apoptotic death in Molt-4 leukemia cells [60], induces G2/M arrest and apoptosis via p38 MAPK inhibition in myeloma cells [37], and decreases breast cancer cell adhesion, migration, and invasion via FAK/Src inhibition [39]. Carvone shows multi-targeted effects against non-small cell lung cancer [61], reduced viability of neuroblastoma cells [62], and displayed chemopreventive effects in DMBA-induced skin tumorigenesis in mice [63]. However, the NCI-60 screen found carvone inactive across 55 cell lines (ChEMBL), indicating that cytotoxicity requires high concentrations or is cell-line specific. This counter-evidence should temper enthusiasm for carvone as an anticancer agent (Table 7).

### 3.4. Neurological and Analgesic Activity

*R*-carvone shows antinociceptive activity in mice with a non-opioid mechanism, and it reduces compound action potential amplitude by ~50% in isolated rat sciatic nerve [27,28]. *S*-carvone shows anticonvulsant-like activity [64] and neuroprotective effects [22,65,66,67,68]. Both enantiomers exhibit antimanic-like effects [66]. (−)-Carvone inhibits oxytocin-induced writhing.

Carvone also modulates TRP channels [69], and both enantiomers enhance spontaneous *L*-glutamate release in the spinal dorsal horn [29]. The GABA-A receptor negative modulation is the primary mechanism underlying these neurological effects. *S*-carvone’s anesthetic activity involves multi-target modulation of GABA-A, NMDA, and Nav1.2 channels [26,35,36] (Table 8).

### 3.5. Cytoprotective Activity

Carvone exhibits modest direct radical scavenging but potent indirect antioxidant activity through the Nrf2/ARE pathway. *R*-carvone induces nuclear Nrf2 translocation and HO-1 induction at 665 µM [17]. *S*-carvone inhibits TBARS-induced oxidative stress [70]. The weak direct scavenging is consistent with carvone’s lack of phenolic hydroxyl groups; the primary antioxidant mechanism is transcriptional activation of ARE-driven cytoprotective genes (HO-1, NQO1, GCLC) [17]. *R*-carvone attenuates doxorubicin-induced cardiotoxicity in vivo while potentiating its anticancer effects in vitro [71]. Carvone also attenuates cardiac fibrosis and inflammation via GSK-3β/PKC-α signaling [38], modulates intracellular calcium signaling, and exhibits antiarrhythmic activity [30] (Table 9).

Hepatoprotective effects include amelioration of ischemia-reperfusion injury [72], attenuation of CCl_4_-induced liver fibrosis via TGF-β/Smad inhibition [40], and suppression of oxidative stress in immobilization stress-induced liver injury [73]. The mechanisms involve antioxidant effects (reducing MDA, increasing GSH), anti-fibrotic effects (collagen deposition reduction), and anti-inflammatory effects (NF-κB-mediated cytokine reduction). Carvone shows nephroprotective effects across multiple acute kidney injury (AKI) models. Studies demonstrate protection against renal ischemia-reperfusion injury [74,77], mitigation of renal damage with enhanced antioxidant defense [38,75], alleviation of LPS-induced AKI via MyD88-dependent signaling [18,78], nephroprotection in sepsis-induced AKI [76,79], and protection against myoglobinuric acute tubular injury via NF-κB/caspase-3 pathway inhibition [14,19]. The mechanisms converge on anti-inflammatory (TLR4/MyD88/NF-κB), antioxidant (Nrf2/HO-1), and anti-apoptotic (caspase-3) effects.

### 3.6. Metabolic Activity

Carvone shows hypoglycemic and hypolipidemic effects in diabetic rat models [15,70,80,81]. It promotes glucose-stimulated insulin secretion via the olfactory receptor Olfr1259 in pancreatic β-TC6 cells [32]. *S*-carvone activates SIRT1, which may contribute to metabolic regulation [16]. In addition, *S*-carvone restrains high-fat diet-induced obesity in mice [82]. Carvone ameliorates polycystic ovary syndrome (PCOS) via SREBP1/TLR4 pathway modulation [20], and inhibits melanogenesis via cAMP/PKA pathway inhibition in B16F10 cells [41].

Both carvone enantiomers show spasmolytic activity across multiple smooth muscle types [69,83]. Carvone and limonene enantiomers relax carbachol-induced contractions in guinea pig trachea and rat aorta, with no significant enantiomer difference [84]. *R*-Carvone has antispasmodic effects on guinea pig ileum [77]. Carvone relaxes arsenic- and mercury-induced aortic hypercontraction [85,86]. The mechanism likely involves calcium channel blockade and/or potassium channel activation, reducing intracellular Ca^2+^ and preventing contraction [30] (Table 10).

### 3.7. Pesticidal and Antiparasitic Activity

*R*-carvone is a potent natural insecticide (contact, fumigant, repellent), e.g., against *Tribolium castaneum*, [12,13], *Sitophilus oryzae*, *Rhyzopertha dominica*, *Spodoptera littoralis*, (Table 11). An aphidicidal nanoemulsion with AChE inhibition has been developed against *Rhopalosiphum maidis* and *Sitobion avenae* [87]. Nematicidal activity against *Meloidogyne incognita* [88] and Molluscicidal activity against *Biomphalaria alexandrina* and *Theba pisana* have been demonstrated. Nanoliposomal carvone formulations show larvicidal activity [89], and carvone combinations show enhanced toxicity against insect pests [90]. Antiparasitic activity includes lethal effects on protoscolices [91], anthelmintic activity of *R*-carvone nanoemulsion [33], and gastrointestinal nematode control [78,92,93]. The insecticidal mechanism involves AChE inhibition and GABA-A receptor negative modulation, both established insecticide targets.

Additional documented activities include anti-arthritic effects against complete Freund’s adjuvant-induced arthritis [79], chemopreventive effects during DMBA-induced skin tumorigenesis [63], immunomodulatory activity [94,95], allelopathic activity via microtubule targeting [42], and gut microbiota modulation [96,97]. Contact allergy and safety data are discussed in ongoing sections.

## 4. Structure–Activity Relationships and Enantiomer Considerations

The carvone structure contains three key functional elements: (i) the α,β-unsaturated ketone (enone system, Michael acceptor potential), (ii) the isopropenyl group (lipophilicity, membrane interaction, stereocenter), and (iii) the C2 methyl group (lipophilicity, membrane interaction). The enone system is critical for AhR antagonism—SAR studies show that carbonyl position and C3/C5-alkylation are structural determinants of antagonist potency [6,7]. The keto group and lipophilic backbone contribute to CYP inhibition potential. The stereocenter drives enantiomer-selective interactions with GABA-A, OR1A1, and NO inhibition, all confirmed by head-to-head testing. Regarding enantiomer comparisons, it is important to distinguish between findings from head-to-head studies (both enantiomers tested in the same assay) and single-enantiomer studies (only one enantiomer tested). Several targets were studied with only one enantiomer: SIRT1 was tested only with *S*-carvone [16], while Nrf2/HO-1 and JNK1 were tested only with *R*-carvone [17]. These single-enantiomer findings should not be interpreted as enantiomer-specific without direct comparison. Properly justified head-to-head differences include GABA-A modulation (*R* > *S*), NO inhibition (*S* > *R*), anti-allergic airway activity (*R* >> *S*), OR1A1 activation ((*S R*)), and insecticidal activity (*R* >> *S*). AhR antagonism, anti-MRSA activity, and spasmolytic activity appear shared (equipotent) between enantiomers.

Carvone’s target engagement concentrations span a wide range, but only a handful of values come from validated assays. The only genuinely active PubChem qHTS result is CYP2D6 inhibition. AhR antagonism is documented by dose–response K_i_ determination and in vivo confirmation. AChE inhibition (K_i_ = 300 µM) is literature-reported but flagged by PubChem as “outside typical range.” The remaining qHTS-derived potency values (TRβ, ADRB2, RORγ, CAR, ARE/Nrf2, ERα, RGS4) come from inconclusive or artifact-prone assays and should not be treated as reliable potencies; they are retained in Table 1 only as screening flags for potential follow-up. High micromolar concentrations are required for SIRT1, JNK1/Nrf2, and GABA-A, which may exceed achievable plasma levels from dietary exposure, though topical application (AhR) and inhalation (insecticidal) bypass first-pass metabolism.

## 5. Computational Clustering Analysis (Exploratory)

The following clustering analysis is exploratory and hypothesis-generating in nature. Silhouette scores of 0.17–0.19 indicate weak partition structure, and the clustering is driven primarily by categorical features (target class, activity mode, evidence tier) rather than pharmacological potency, as only one target entry (AhR K_i_) carries a genuine literature potency value. The results should be interpreted as descriptive summaries of the dataset structure, not as statistically validated groupings. To identify natural groupings among carvone’s targets and activities, Ward’s hierarchical clustering (Euclidean distance) was performed on standardized feature matrices. Features included one-hot encoded target class, activity mode, enantiomer group, log_10_-transformed potency, and evidence tier (A = 3, B = 2, C = 1, D = 0). The potency feature was restricted to true IC_50_/EC_50_/K_i_ values from peer-reviewed literature; percentage readouts, tested concentrations, GABA-A substrate-EC_50_ shift values, qHTS curve-fit AC_50_ values, and database-deposited potencies (including the ChEMBL-sourced AChE K_i_) were excluded. Only 1 of 49 target entries carries a true literature potency value (AhR K_i_ = 11–48 µM); the remaining entries have missing potency, imputed to this single value. Consequently, clustering is driven primarily by categorical features and the evidence tier.

Target clustering (k = 5, silhouette = 0.17) separated targets by class, activity mode, and evidence tier: Cluster 1 (six members) contains nuclear/other receptors with agonist mode; Cluster 2 (19 members) contains ion channels and the high-evidence targets, including AhR (Tier A × 3), SIRT1, Nrf2, GABA-A, and TRPV1—the most robustly characterized targets cluster together, reflecting their multi-assay validation; Cluster 3 (six members) contains pathogen/pest targets; Cluster 4 (12 members) contains signaling pathways with inhibitor mode, including most Tier D artifacts (ADRB2, TRβ, ERα, ALDH1A1, Menin-MLL); and Cluster 5 (six members) contains kinases and signaling pathways (JNK1, p38 MAPK, NF-κB/p65).

**Figure 2:**
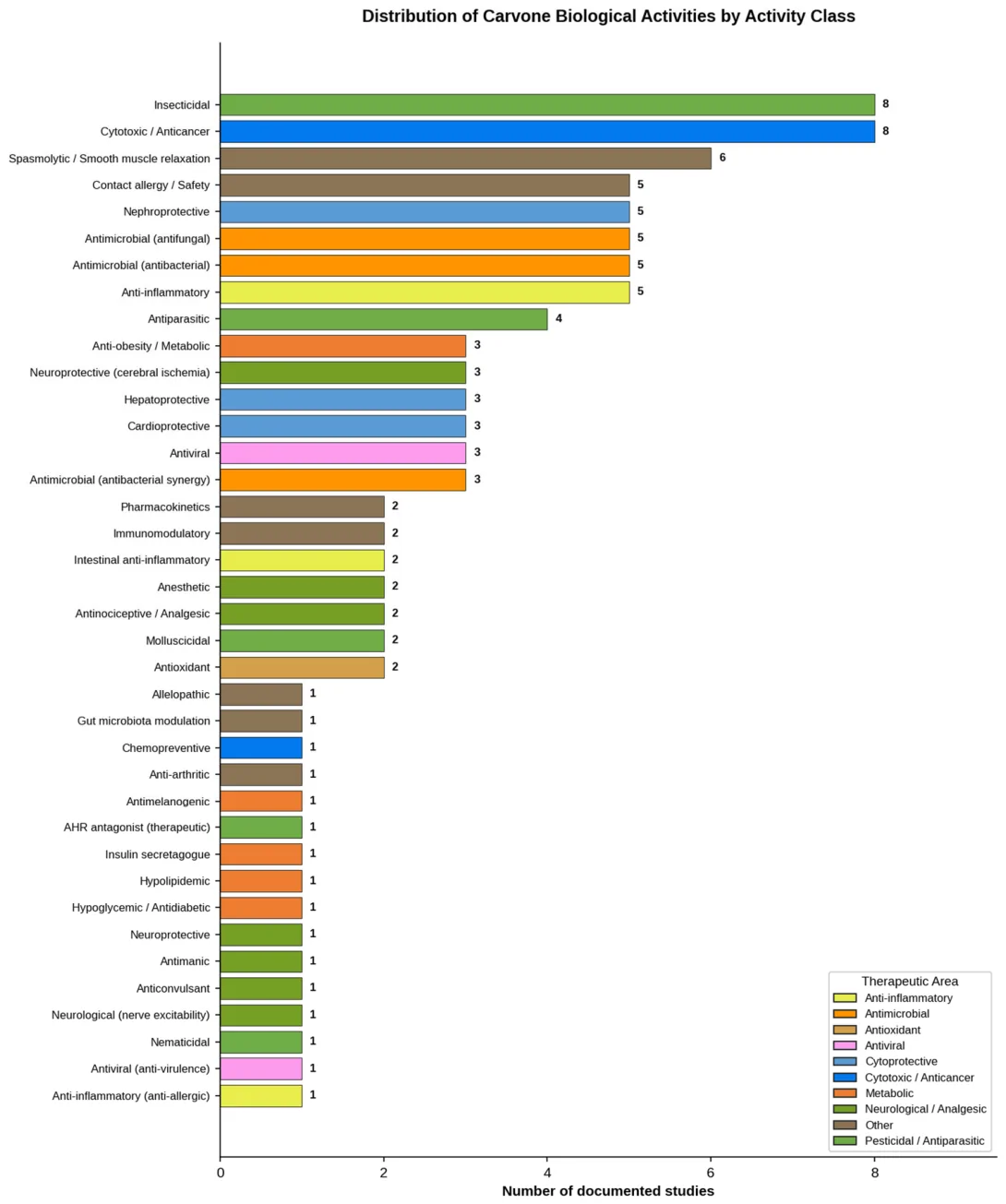
Distribution of carvone biological activities. Displayed across 38 activity classes, color-coded by 10 therapeutic areas. Bar length indicates the number of documented studies within each activity class.

Activity clustering (k = 6, silhouette = 0.19) was performed on the 99 documented activities (excluding the ARE/Nrf2 qHTS artifact) and separated activities by therapeutic area and evidence tier: Cluster 3 (28 members, dominant Tier A/B) contains all five Tier A nephroprotective activities plus other cytoprotective in vivo studies; Cluster 1 (nine members, dominant Tier C) contains antimicrobial screening-level activities; and the remaining clusters group activities by therapeutic area with tier distributions reflecting evidence quality. The silhouette scores indicate that the chosen partitions are not statistically optimal but were selected for biological interpretability. Because the clustering was re-implemented on the corrected post-audit data with revised categorical encodings and the evidence-tier feature replacing the former confidence grade, the change in silhouette scores relative to the earlier confidence-based analysis (target 0.20 → 0.17; activity 0.10 → 0.19) reflects the combined effect of these changes and cannot be attributed to the tier feature alone. Figure 2 shows the distribution of biological activities across all 38 classes.

## 6. Safety and Contact Allergy

Carvone is a recognized contact allergen, primarily associated with spearmint oil exposure, and there is a safety consideration directly relevant to the traditional topical and cosmetic uses of carvone-rich essential oils. A 21-year study in southern Sweden documented the prevalence of carvone contact allergy [98], and the North American Contact Dermatitis Group has reported patch testing results [99]. Late-appearing patch test reactions to carvone have been investigated [100], and oral lichenoid lesions associated with carvone contact allergy have been reported in snuff users [101]. Consecutive patch testing studies have examined the use of carvone in clinical practice [102]. The antigen formation mechanism of carvone and related terpenes has been characterized [103], and carvone has been identified as an overlooked contact allergen cross-reacting with sesquiterpene lactones [104]. Use tests with *R*-carvone in toothpaste on sensitized individuals have been performed [105].

Regarding genotoxicity, Nesterkina et al. evaluated the genotoxic and mutational potential of monocyclic terpenes, including carvone [106]. The NTP conducted toxicology and carcinogenesis studies of carvone. Pharmacokinetic studies show that carvone undergoes stereoselective metabolism. Percutaneous absorption studies implicate stereoselective metabolism in determining blood levels [3], and oral pharmacokinetics of carvone have been characterized alongside menthol [107]. These PK data are relevant to the traditional exposure routes—dietary (enteric first-pass), topical (percutaneous, relevant to the carminative and cosmetic uses), and inhalation from essential oils—and to the high micromolar concentrations required for some in vitro targets, which may exceed achievable plasma levels from dietary exposure, though local tissue concentrations at the application site (skin, gastrointestinal tract) are likely substantially higher.

## 7. Discussion

### 7.1. Physicochemical Considerations and Assay Interference

Carvone is a low-molecular-weight (MW 150.22), volatile, lipophilic monoterpene ketone with limited aqueous solubility (approximately 0.6 g/L). These physicochemical properties give rise to several technical challenges that should be considered when interpreting the in vitro and in vivo evidence compiled in this review. First, carvone’s volatility means that effective concentrations in cell culture experiments may be lower than nominal concentrations due to evaporation, particularly in open systems or during prolonged incubation. Second, at the high micromolar concentrations required for many targets (e.g., SIRT1 at 265 µM, JNK1/Nrf2 at 665 µM, GABA-A at 90 µM), nonspecific membrane interactions and detergent-like effects may contribute to observed bioactivity independently of specific target engagement. Third, adsorption to plastic surfaces (plates, tubes) may reduce free concentrations in assay systems. Fourth, the α,β-unsaturated ketone (enone) moiety of carvone is a potential Michael acceptor, raising the possibility of covalent modification of cysteine residues on proteins, which could produce apparent activity through nonspecific reactivity rather than selective target binding. While the AhR antagonism is supported by competitive binding studies and in vivo validation at pharmacologically relevant concentrations, the high micromolar potency values for other targets should be interpreted with caution in light of these physicochemical considerations.

### 7.2. Limitations

Several limitations of this review should be acknowledged. (a) Publication bias: Positive results are overrepresented in the literature, and negative findings (particularly from qHTS screens) may be underreported. We have attempted to mitigate this by explicitly including inactive and inconclusive database results. (b) Database incompleteness: PubChem and ChEMBL coverage is not exhaustive, and these databases are continuously updated. The assay counts reported here reflect the state of the databases as of 14 July 2026. (c) Stereochemical ambiguity: Many studies do not specify which enantiomer was used, or employ racemic material, precluding conclusions about enantiomer selectivity. Where enantiomer identity is specified in the original report, it is noted in our tables. (d) Assay heterogeneity: different protocols, cell lines, endpoints, and concentration ranges across studies make direct cross-study comparison difficult, and the evidence-tiering framework represents an attempt to stratify rather than standardize these diverse data. (e) Lack of clinical evidence: no human clinical trials of carvone for any therapeutic indication have been reported; all pharmacological evidence is preclinical. (f) High micromolar concentrations: the concentrations required for many targets may exceed achievable plasma levels from dietary exposure, and the relevance of in vitro findings to in vivo pharmacology remains to be established.

### 7.3. Ethnopharmacological Mapping

The compiled mechanistic evidence provides a plausible mechanistic basis for several of carvone’s traditional uses, though it should be emphasized that traditional use can motivate a pharmacological hypothesis but does not by itself validate a target mechanism. The carminative and digestive applications of carvone-rich plants (spearmint, caraway, dill) [1,2,4] may be consistent with carvone’s modulation of gastrointestinal smooth muscle (spasmolytic activity via calcium channel blockade) and GABA-A receptor negative modulation, both of which would reduce gastrointestinal hypermotility and spasm. The antimicrobial and food-preservative traditional uses are consistent with the broad-spectrum antimicrobial activity and quorum-sensing inhibition. The traditional topical uses—including spearmint oil in cosmetics and carvone as a transdermal skin permeabilizer—may be consistent with the AhR antagonism validated in mouse skin, while the contact-allergy findings represent the corresponding safety boundary of these topical applications. The anti-inflammatory traditional uses are consistent with the SIRT1/NF-κB and Nrf2/HO-1 pathways. However, carvone-rich essential oils contain multiple bioactive constituents, and the observed effects of whole-plant preparations cannot be attributed solely to carvone. Clinical validation of these traditional-use applications—particularly the carminative/digestive and topical indications—represents the most direct translational path for carvone.

## 8. Materials and Methods

### 8.1. Literature Search and Corpus Assembly

This systematic review was conducted in accordance with the Preferred Reporting Items for Systematic Reviews and Meta-Analyses (PRISMA) 2020 statement. A completed PRISMA 2020 checklist is provided as Supplementary Materials. The review was not registered in a prospective systematic review register (e.g., PROSPERO) because it concerns molecular target characterization and bioactivity profiling of a natural product rather than evaluation of a clinical intervention. No review protocol was prepared prior to conducting the review; the systematic search strategy is fully described below.

**Figure 3:**
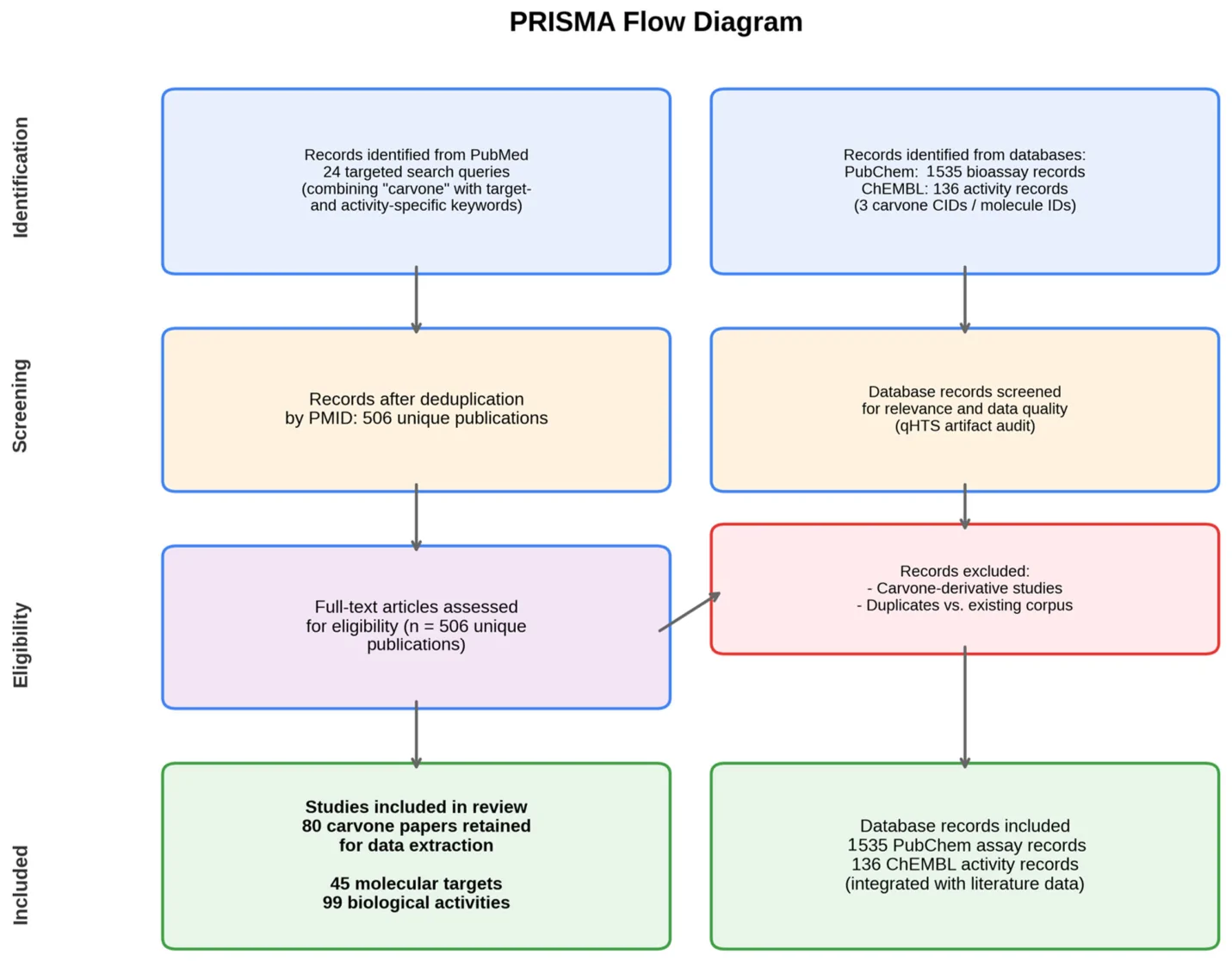
PRISMA flow diagram showing the literature and database record identification, screening, eligibility, and inclusion process.

The literature search covered publications indexed in PubMed from inception through July 2026. A comprehensive PubMed search was conducted using the NCBI E-utilities API (Figure 3) (esearch and efetch) with 24 targeted queries combining the term “carvone” with target- and activity-specific keywords. Queries were designed to capture both enantiomers and the racemic form. The search retrieved 506 unique publications (deduplicated by PMID). After removing duplicates against the existing report corpus and excluding carvone-derivative studies, 80 truly new carvone papers were retained for data extraction. The scope was restricted to carvone itself to maintain chemical specificity.

### 8.2. Data Extraction

For each retained paper, molecular target entries and biological activity entries were extracted into structured tables. Target entries capture target name, target identifier (UniProt/Gene), activity type (agonist, antagonist, inhibitor, activator, modulator), activity measure and value (K_i_, IC_50_, EC_50_, K_d_, or percentage effect), units, enantiomer (*S*, *R*, both, or unspecified), species/model system, assay type, source citation, and a confidence grade (High, Medium, Low). Activity entries capture activity class, specific activity, model system, quantifier and value, enantiomer, species, source citation, and confidence grade. Each entry was tagged with its literature citation number or, for database-only records, the PubChem AID or ChEMBL identifier. Molecular targets and biological activities are counted as separate categories. A single study may contribute entries to both the target table and the activity table. Within each category, entries represent distinct target–assay or activity–model combinations; the same observation is not counted multiple times within a category. The 45 targets and 99 activities are thus non-overlapping within their respective categories but may derive from the same underlying studies.

### 8.3. PubChem and ChEMBL Data Retrieval

PubChem bioassay data for the three carvone CIDs (16,724 = *S*-carvone, 439,570 = *R*-carvone, 7439 = racemic) were retrieved via the PubChem REST API (pug/assay/aid/AID/CSV). A total of 1535 assay records were retrieved across the three CIDs. ChEMBL activity data were retrieved via the ChEMBL API (chembl/api/data/activity.json) for the three carvone molecule IDs (CHEMBL501949 = *S*-carvone, CHEMBL2229268 = *R*-carvone, CHEMBL15676 = unspecified), yielding 136 activity records. PubChem and ChEMBL assay identifiers are cited by ID number within the tables and are not included in the numbered reference list, as they are database records rather than publications. PubChem bioassay data were retrieved on 14 July 2026. ChEMBL activity data were retrieved on 14 July 2026. As these databases are continuously updated, assay counts and outcomes may differ from subsequent accesses.

### 8.4. qHTS Data-Quality Audit

Because PubChem and ChEMBL qHTS assays report curve-fit potency values even for inconclusive or artifact-affected results, a systematic data-quality audit was performed on all database-sourced entries in both the target table and the activity table. For each PubChem AID, the raw assay CSV was parsed to extract the activity outcome (Active, Inactive, Inconclusive), activity score, phenotype classification, and curve class. For each ChEMBL target, the activity records were queried to extract the activity comment field (Inconclusive, Not Active, Active) and the assay description. Entries were classified as (i) validated (genuinely Active outcome with high activity score), (ii) inconclusive (Inconclusive outcome, retained as low-confidence screening flags), or (iii) artifact (Inconclusive outcome with a phenotype that contradicts the assay design—e.g., cytotoxic phenotype in a receptor assay, fluorescent phenotype indicating readout interference, or inhibitor phenotype in an agonist-design assay). Artifact entries were relabeled with the specific artifact reason, and their curve-fit potency values were removed.

### 8.5. Reference Metadata Verification

Reference metadata (authors, title, journal, year, volume, issue, pages, DOI) were verified via CrossRef (api.crossref.org/works/DOI) and PubMed E-utilities (efetch with db = pubmed). References were sorted by first author surname and numbered. In-text citations and table citations use the numeric reference; PubChem and ChEMBL assay IDs are cited by identifier within tables only.

### 8.6. Computational Clustering

To identify natural groupings among carvone’s targets and activities, Ward’s hierarchical clustering (Euclidean distance) was performed on standardized feature matrices using scipy.cluster.hierarchy. Features included one-hot encoded target class, activity mode, enantiomer group, log_10_-transformed potency, and evidence tier (A = 3, B = 2, C = 1, D = 0; see Section 8.7). The potency feature was restricted to true IC_50_/EC_50_/K_i_ values from peer-reviewed literature; percentage readouts, tested concentrations, GABA-A substrate-EC_50_ shift values, qHTS curve-fit AC_50_ values, and database-deposited potencies (including the ChEMBL-sourced AChE K_i_ of 300 µM) were excluded. The number of clusters (k) was selected for biological interpretability rather than statistical optimality, and silhouette scores were reported as a measure of partition quality. Clustering was performed on the corrected post-audit data with evidence tiers assigned, resolving the earlier limitation of clustering on pre-audit data.

### 8.7. Evidence-Tiering Framework

To stratify targets and activities by the robustness of their supporting evidence, a four-tier grading system (A/B/C/D) was applied to every entry. The framework distinguishes multi-endpoint orthogonal characterization from single-concentration screening flags and computational predictions, preventing over-interpretation of low-evidence hits.

Tier A—Comprehensive characterization. Requires ≥ 2 distinct assay types AND in vivo validation AND (≥2 endpoints OR ≥ 3 orthogonal methods). The in vivo-plus-multi-endpoint requirement was set to reserve Tier A for targets with genuinely multi-dimensional characterization rather than any single in vivo study; this threshold was calibrated against the dataset to yield a small, defensible Tier A set.

Tier B—Solid single-method characterization. Requires dose–response quantification (K_i_/IC_50_/EC_50_ or % effect at specified concentration) from peer-reviewed literature OR in vivo validation with a primary endpoint OR a comprehensive screening panel with dose–response quantification (for inactives, including ceiling-censored panels where all values exceed the assay’s upper limit).

Tier C—Screening flag. qHTS or single-concentration screening without independent dose–response or in vivo validation. Includes both genuinely “active” qHTS hits and inconclusive qHTS hits. Retained as potential follow-up targets, not established interactions.

Tier D—Weak/unverified. Artifact-flagged entries, in silico-only predictions, or unverifiable records. Tier D overrides other criteria.

For targets, six grading dimensions were computed: (1) endpoint count, (2) assay type diversity, (3) dose–response characterization, (4) in vivo validation, (5) orthogonal method count, and (6) independent replication.

For activities, four dimensions were computed: (1) model system level, (2) endpoint count, (3) dose–response, and (4) replication.

Tier assignment is rule-based rather than a pure score sum, because combinations matter (e.g., Tier A requires multi-assay AND in vivo, not just a high score). Inactive entries receive the same A/B/C/D tier using adapted criteria; because negative results from in vitro panels do not require in vivo validation to be informative, the Tier A in vivo requirement is replaced by an equivalent breadth criterion.

## 9. Conclusions

Carvone is a multi-target bioactive monoterpene whose pharmacological profile extends well beyond its traditional uses as a flavoring and fragrance compound. This review documents 45 distinct molecular targets and 99 documented biological activities, supported by literature references, with each entry stratified by a four-tier evidence framework (A/B/C/D) that distinguishes comprehensive multi-assay characterization from screening flags and artifacts. The aryl hydrocarbon receptor stands out as the most robustly characterized Tier A target by the evidence-tiering criteria; both enantiomers act as noncompetitive allosteric antagonists at pharmacologically relevant concentrations, validated in vivo, and extended by recent SAR studies [5,6,7]. The GABA-A receptor also achieves Tier A status through multi-assay characterization with convergent in vivo behavioral evidence (see itemized justification, Table 3 footnote). This designation reflects the breadth and quality of evidence for AhR antagonism specifically, not a claim that AhR mediates all of carvone’s traditional or pharmacological effects. The Tier B targets—including JNK1, SIRT1, Nrf2, TRPV1, AChE, and OR1A1—rest on solid dose–response or in vivo evidence, while the Tier C targets (CYP2D6, CYP1A2, RORγ, CAR, TDP-43, and others) are qHTS screening flags that require confirmatory study before being considered established interactions. The Tier D entries are artifacts, in silico-only predictions, or unverifiable records and should not be interpreted as carvone targets.

The breadth of carvone’s cytoprotective activities—spanning anti-inflammatory, cardioprotective, hepatoprotective, and nephroprotective effects—is remarkable for a dietary monoterpene and is mechanistically linked to Nrf2/ARE activation and NF-κB inhibition. The Tier A nephroprotective activities represent the highest-evidence activity cluster, each validated in vivo with multiple endpoints and dose–response quantification. The antimicrobial and pesticidal activities, mediated by membrane disruption and nervous system target modulation (AChE, GABA-A), support carvone’s potential as a natural preservative and pest control agent.

The NCI-60 screen (Tier B) found carvone inactive across all 55 cancer cell lines (GI_50_ > 100 µM), contrasting with individual cytotoxicity reports and suggesting that anticancer activity is cell-line-specific or requires high concentrations. The counterscreen (Tier A) individually tested steroid/nuclear receptors (AR, ERα, ERβ, PR, GR, RXRα, PPARγ—all inactive by radioligand binding), 468 human protein kinases (467/468 above the inhibition cutoff, with only the TK2 pseudokinase marginally affected), the phosphatases PTPN11/SHP2 and PTPN6/SHP1 (inactive), and HSP90 (no geldanamycin displacement), establishing that carvone’s target profile is selective rather than promiscuous and that its AhR antagonism is not mediated by steroid/nuclear receptor binding, promiscuous kinase inhibition, or HSP90 chaperone disruption [5]. Database inactives from PubChem and ChEMBL (Tier C) document single-assay negative results for CYP2D6, CYP1A2, TRβ, ADRB2, ALDH1A1, and *Plasmodium falciparum*.

Several challenges temper these conclusions. The high micromolar concentrations required for some targets (SIRT1, JNK1/Nrf2, GABA-A) may exceed achievable plasma levels from dietary exposure. Many targets were studied with only one enantiomer, precluding conclusions about enantiomer selectivity. Future research should prioritize head-to-head enantiomer comparisons on key targets, clinical pharmacokinetic studies, confirmatory dose–response assays for Tier C screening flags, and translational investigation of AhR antagonism, which currently represents the most promising therapeutic lead [108].

From an ethnopharmacological perspective, the compiled mechanistic evidence provides a plausible mechanistic basis for several of carvone’s traditional uses (see Section 7.3 for detailed discussion). Clinical validation of these traditional-use applications—particularly the carminative/digestive and topical indications—represents the most direct translational path for carvone.

## Figures and Tables

**Table 2:** Molecular targets of carvone: Enzymes. Sub-rows represent distinct assays, measures, or studies. PubChem and ChEMBL assay IDs are cited in the Ref column and are not included in the reference list. Evidence tier: A = comprehensive characterization; B = solid single method; C = screening flag; D = weak/unverified.

Target	Activity Type	Measure/Value	Enantiomer	Species/Model	Ref	Tier
Acetylcholinesterase (AChE)	Inhibitor (mixed-type, reversible; deposited via ChEMBL)	K_i_ (flagged “outside typical range” by PubChem): 300 µM	*S*	Electric eel (*Electrophorus electricus*)	PubChem AID 1098346	C
Aldehyde dehydrogenase 1A1 ALDH1A1	Inhibitor (qHTS, INCONCLUSIVE)	Curve-fit values from inconclusive records	*S*	Human (biochemical)	ChEMBL CHEMBL3577	D
Cytochrome P450 (Bx-cyp29A3, nematode)	Inducer (stress response)	Gene expression (PCR): Active (stress-induced expression)	*S*	Nematode (*Bursaphelenchus xylophilus*)	[23]	C
Cytochrome P450 1A2 (CYP1A2)	Inhibitor (qHTS, INCONCLUSIVE)	Curve-fit AC_50_ (inconclusive)	*S*, *R*	Human (luciferase reporter)	PubChem AID 1671199	C
Cytochrome P450 2D6 (CYP2D6)	Inhibitor	Potency (qHTS)	*S*	Human (luciferase reporter)	PubChem AID 1671196	C
Matrix metalloproteinase-9 (MMP9, in silico)	Inhibitor (in silico docking)	Binding energy (molecular dynamics): (docking score)	*R*	Human (in silico)	[24]	D

**Table 4:** Molecular targets of carvone: Kinase and signaling pathways. Sub-rows represent distinct assays, measures, or studies. PubChem and ChEMBL assay IDs are cited in the Ref column and are not included in the reference list. Evidence tier: A = comprehensive characterization; B = solid single method; C = screening flag; D = weak/unverified.

Target	Activity Type	Measure/Value	Enantiomer	Species/Model	Ref	Tier
FAK/Src signaling pathway (breast cancer)	Inhibitor (FAK activation)	Protein phosphorylation (WB): Active, dose-dependent	*S*	Human breast cancer cells, Mouse Ehrlich carcinoma	[39]	B
GSK-3β/PKC-α signaling pathway (cardiac)	Modulator (pathway)	Protein expression (WB): (significant)	Unspecified	Rat PCOS-induced cardiac fibrosis	[38]	B
TGF-β/Smad signaling (liver fibrosis)	Inhibitor (pathway)	Protein expression (IHC, WB): (significant)	*S*	Rat CCl4-induced liver fibrosis	[40]	B
c-Jun N-terminal kinase 1 (JNK1/MAPK8)	phosphorylation inhibition	decrease in JNK1 phosphorylation	*S*	Mouse RAW264.7 macrophages	[16]	B
c-Jun N-terminal kinase 1 (JNK1/MAPK8)	phosphorylation inhibition	decrease in JNK1 phosphorylation	*R*	Mouse RAW264.7 macrophages	[17]	B
cAMP/PKA pathway (melanogenesis)	Inhibitor (cAMP/PKA)	cAMP levels, melanin: Active, dose-dependent	Unspecified	Human B16F10 melanoma cells	[41]	C
p38 MAPK	Inhibitor (pathway)	Inhibition of p38 MAPK: Not quantified	Unspecified	Human myeloma cells	[37]	C
Microtubules (tubulin)	Inhibitor (depolymerization/allelopathic)	Root growth inhibition (gas phase): Active	*R*	Plant (*Arabidopsis thaliana*, Poppy, Cress)	[42]	C
Quorum sensing (CviR/CviI system)	Inhibitor (QS)	Violacein inhibition, biofilm reduction	Unspecified	*Chromobacterium violaceum*	[43]	C
Quorum sensing (CviR/CviI system)	Inhibitor (QS)	biofilm reduction	*S*	*Hafnia alvei*	[44]	C

**Table 5:** Biological activities of carvone: Anti-inflammatory activity. Sub-rows represent individual studies. PubChem and ChEMBL assay IDs are cited in the Ref column and are not included in the reference list. Tier: A = comprehensive characterization; B = solid single method; C = screening flag; D = weak/unverified.

Activity Class	Specific Activity	Model System	Quantifier/Value	Enantiomer	Ref	Tier
Anti-inflammatory	Inhibition of LPS-induced NO production (NOS2 expression)	RAW264.7 murine macrophages	NO production; *S* > *R* in potency	*S*, *R*	[45]	B
Anti-inflammatory	Decreased IL-1β mRNA and protein	RAW264.7 murine macrophages + primary human chondrocytes	Significant decrease	*S*, *R*	[45]	B
Anti-inflammatory	Inhibition of NF-κB transcriptional activity (via SIRT1 activation → p65 deacetylation)	RAW264.7 macrophages	Significant decrease in Ac-p65(Lys310)	*S*	[16]	B
Anti-inflammatory	Inhibition of NF-κB (via JNK1 inhibition + Nrf2 activation → decreased CBP/p300 availability)	RAW264.7 murine macrophages	Significant HO-1 increased	*R*	[17]	B
Anti-inflammatory (anti-allergic)	Inhibition of eosinophil infiltration, mucus production, increase IL-10	BALB/c mice, OVA-induced allergic airway inflammation	*R* >> *S*	*S*, *R*	[46]	B
Intestinal anti-inflammatory	Protection against DSS-induced ulcerative colitis	BALB/c mice, DSS-induced UC	Colon length, histology, inflammatory markers Active	*S*	[47]	B
Intestinal anti-inflammatory	Gastroprotective effect (*Aloysia polystachya* extract)	Mouse, ethanol-induced gastric ulcer)	Ulcer index + gastric histology: Active (dose-dependent)	Unspecified (carvone from *A. polystachya*)	[48]	B

**Table 6:** Biological activities of carvone: Antimicrobial activity. Sub-rows represent individual studies. PubChem and ChEMBL assay IDs are cited in the Ref column and are not included in the reference list. Tier: A = comprehensive characterization; B = solid single method; C = screening flag; D = weak/unverified.

Activity Class	Specific Activity	Model System	Quantifier/Value	Enantiomer	Ref	Tier
Antibacterial synergy	Synergistic with gentamicin	MRSA strains (checkerboard assay)	FICI	*S*, *R*	[8]	C
Antibacterial synergy	Synergy with colistin	Colistin-resistant *P. aeruginosa*	FICI + time-kill curves: Synergistic	Unspecified (carvone)	[9]	C
Antibacterial synergy	Synergy with antibiotics	Antibiotic-resistant bacteria	FICI + MIC reduction: Synergistic	*R*	[10]	C
Antibacterial	Anti-MRSA activity (MIC)	6 *S. aureus* strains	MIC	*S*, *R*	[8]	B
Antibacterial	Antibacterial + antibiofilm	*Staphylococcus aureus*	Not quantified (qualitative activity)	Unspecified	[11]	B
Antibacterial	wound healing	MRSA + diabetic wound model	Effective, MIC	*S*	[51]	B
Antibacterial	Quorum sensing inhibition + biofilm blockade	*Chromobacterium. violaceum* ATCC 12472	Violacein inhibition + biofilm reduction	Unspecified (carvone)	[43]	B
Antibacterial	Antibacterial creams	Various bacteria (cream formulations)	Growth inhibition zones: Active	*S*	[56]	B
Antifungal	Anticandidal activity (inhibition of yeast-to-filament transition)	*Candida albicans* H317	Active; growth inhibition	*S*	ChEMBL ID 90–92; [49]	C
Antifungal	Antifungal synergy with itraconazole/caspofungin	*Aspergillus fumigatus*	Not quantified (synergy study)	Unspecified	[55]	C
Antifungal	Antifungal (cell membrane disruption)	*Botryosphaeria dothidea*	MIC	Unspecified	[52]	C
Antifungal	Antifungal	*Malassezia* spp. (clinical isolates)	MIC + MFC: Active	Unspecified (carvone)	[53]	C
Antifungal	Inhibition of filamentation	*C. albicans*	Filamentation inhibition: Active	Unspecified (carvone)	[54]	C
Antiviral	Inhibition of replication	Vero 76 cells infected with SARS-CoV-2	EC_50_, IC_50_	Unspecified	[57]	B
Antiviral	Anti-Toxoplasma activity	*Toxoplasma gondii* RH in human HFF cell	IC_50_	Unspecified	ChEMBL ID 89	B
Antiviral	Interaction with SARS-CoV-2 spike RBD	In silico (molecular docking)	Binding energy: Predicted binding	Unspecified (carvone)	[58]	B
Anti-virulence	Inhibition of virulence factors and biofilm	*Serratia marcescens*	Not quantified (qualitative)	Unspecified	[50]	B

**Table 7:** Biological activities of carvone: Cytotoxic and anticancer. Sub-rows represent individual studies. PubChem and ChEMBL assay IDs are cited in the Ref column and are not included in the reference list. Tier: A = comprehensive characterization; B = solid single method; C = screening flag; D = weak/unverified.

Activity Class	Specific Activity	Model System	Quantifier/Value	Enantiomer	Ref	Tier
Chemopreventive	Preventive effect; DMBA-induced skin tumorigenesis	Mouse (Swiss albino, DMBA-induced skin cancer)	Tumor incidence + phase I/II enzyme expression: Active	*S*	[63]	B
Cytotoxic/Anticancer	Induction of p53/caspase-3 mediated apoptosis; inhibition of migration	Breast cancer cell lines (MCF-7, MDA-MB-231)	Dose-dependent (IC_50_)	*R*	[59]	B
Cytotoxic/Anticancer	ROS-mediated apoptotic cell death	Molt-4 human leukemic cells	Dose-dependent	*S*	[60]	B
Cytotoxic/Anticancer	Inhibition of proliferation, clonogenicity, wound healing; apoptosis induction	NSCLC cell lines	Dose-dependent	*S*	[61]	B
Cytotoxic/Anticancer	Apoptosis induction, G2/M arrest, inhibition of invasion via p38 MAPK inhibition	Myeloma cells	Dose-dependent	Unspecified	[37]	B
Cytotoxic/Anticancer	NCI-60 screen: inactive	NCI-60 human tumor cell line panel	GI_50_: inactive across all lines	Unspecified	ChEMBL NCI-60	B
Cytotoxic/Anticancer	Reduced cell viability	N2a neuroblastoma + primary rat neurons	Active	Unspecified	[62]	B
Cytotoxic/Anticancer	Decreased breast cancer adhesion, migration, invasion (FAK/Src suppression)	Human breast cancer cells + mouse (Ehrlich carcinoma)	Migration/invasion + tumor growth: Active (dose-dependent)	*S*	[39]	B

**Table 8:** Biological activities of carvone: Neurological and analgesic. Sub-rows represent individual studies. PubChem and ChEMBL assay IDs are cited in the Ref column and are not included in the reference list. Tier: A = comprehensive characterization; B = solid single method; C = screening flag; D = weak/unverified.

Activity Class	Specific Activity	Model System	Quantifier/Value	Enantiomer	Ref	Tier
Anesthetic	Anesthetic activity (GABA-A positive modulation, NMDA + Nav1.2 inhibition)	Rat (in vivo) + Xenopus oocytes (in vitro)	EC_50_ + IC_50_ (channel modulation): Active	*S*	[26]	B
Anesthetic	Analgesic effect	Sheep (intramuscular administration)	PK parameters + analgesic response	*S*	[35,36]	B
Anticonvulsant	Anticonvulsant-like activity (CNS depressant)	Mice	Not quantified (behavioral)	*S* > *R*	[64]	B
Antimanic	Antimanic-like effects (blocking hyperactivity)	Mice, methylphenidate + sleep deprivation	Not quantified (behavioral)	*S*, *R*	[66]	B
Antinociceptive/Analgesic	Antinociceptive activity (acetic acid writhing)	Mice (Swiss, *i.p.* administration)	Effective (similar to morphine)	*R*	[27,28,69]	B
Antinociceptive/Analgesic	Inhibition of formalin-induced nociception	Mice	Effective; non-opioid mechanism	*R*	[27,28]	B
Neurological (nerve excitability)	Reduction of peripheral nerve excitability	Rat isolated sciatic nerve	CAP reduction	*R*	[27,28]	B
Neuroprotective	Neuroprotective + cognitive enhancement	SH-SY5Y cells + mice (in vivo/ex vivo)	improved memory; *S*-enantiomer active, *R* not	*S* (*R* inactive)	[65]	B
Neuroprotective (cerebral ischemia)	Inhibition of cerebral I/R inflammatory response (TLR4/NLRP3)	Rat (cerebral ischemia/reperfusion)	Infarct volume, inflammatory markers, neurological score	*S*	[22]	B
Neuroprotective (cerebral ischemia)	Neuroprotective + cognitive enhancement (Alzheimer model)	Rat (AD-associated dementia model)	Behavioral, neurobiochemical markers: Active (dose-dependent)	Unspecified	[68]	B
Neuroprotective (cerebral ischemia)	Protection: paclitaxel-induced retinal and optic nerve cytotoxicity	Rat (paclitaxel-induced retinotoxicity)	Histopathology + retinal ganglion cell count: Active (protective)	Unspecified	[67]	B

**Table 9:** Biological activities of carvone: Cytoprotective. Sub-rows represent individual studies. PubChem and ChEMBL assay IDs are cited in the Ref column and are not included in the reference list. Tier: A = comprehensive characterization; B = solid single method; C = screening flag; D = weak/unverified.

Activity Class	Specific Activity	Model System	Quantifier/Value	Enantiomer	Ref	Tier
Antioxidant	DPPH radical scavenging activity	Cell-free DPPH assay	EC_50_	Unspecified	ChEMBL ID [17]	B
Antioxidant	Inhibition of TBARS-induced oxy-stress	Rat liver + brain homogenates	Significant inhibition (not quantified)	*S*	[70]	B
Cardioprotective	Attenuation of doxorubicin-induced cardiotoxicity, potentiation of anticancer eff.	Rat (in vivo), MCF-7 cells (in vitro)	Cardiac enzyme levels, tumor cell viability: Active (dose-dependent)	*R*	[71]	B
Cardioprotective	Antiarrhythmic action (calcium signaling modulation)	Rat hearts + ventr. myocytes	Arrhythmia score, ICa,L current	*R*	[30]	B
Cardioprotective	Attenuation of cardiac fibrosis and inflammation, GSK3β/PKCα	Rat, PCOS-induced cardiac fibrosis	Fibrosis markers, cardiac function: significant	Unspecified	[38]	B
Hepatoprotective	Amelioration of hepatic ischemia-reperfusion injury (antiinflammatory, antioxidant)	Rat (hepatic I/R model)	Liver enzymes, histology, inflammatory markers: Active (dose-dependent)	*S*	[72]	B
Hepatoprotective	Attenuation of liver fibrosis (TGF-β/Smad inhibition)	Rat, CCl_4_- induced fibrosis	Fibrosis score, TGF-β/Smad expression: significant	*S*	[40]	B
Hepatoprotective	Suppression of oxidative stress and inflammation in liver (immobilisation stress)	Rat (chronic immobilisation stress)	Liver enzymes + oxidative stress markers: significant	Unspecified	[73]	B
Nephroprotective	Protection against renal ischemia-reperfusion injury	Rat (renal I/R model)	Kidney function markers, histology: significant	*S*	[74]	A
Nephroprotective	Mitigation of Li-induced nephrotoxicity	Rat (Li-induced nephrotoxicity)	Kidney function, oxidative stress markers: Active	*S*	[75]	A
Nephroprotective	Alleviation of LPS-induced AKI (MyD88-dependent pathway)	Mouse (LPS-induced AKI)	Kidney function, inflammatory markers: significant	*S*	[18]	A
Nephroprotective	Nephroprotective in LPS-induced sepsis-associated renal injury	Mouse, sepsis AKI, LPS-induced	Kidney function, inflammatory/apoptotic markers: Active	*S*	[76]	A
Nephroprotective	Protection against myoglobinuric acute tubular necrosis (NF-κB/caspase-3)	Mouse, glycerol-induced AKI	Kidney function, apoptosis markers: significant	*S*	[19]	A

**Table 10:** Biological activities of carvone: Metabolic. Sub-rows represent individual studies. PubChem and ChEMBL assay IDs are cited in the Ref column and are not included in the reference list. Tier: A = comprehensive characterization; B = solid single method; C = screening flag; D = weak/unverified.

Activity Class	Specific Activity	Model System	Quantifier/Value	Enantiomer	Ref	Tier
Anti-obesity/Metabolic	Restraint of high-fat diet-induced obesity, hepatic steatosis, insulin resistance	Mouse (C57BL/6, high-fat diet)	Body weight and composition, glucose and insulin tolerance: Active	*S*	[82]	B
Anti-obesity/Metabolic	Regulation of lipid mobilization and adipogenesis	3T3-L1 adipocytes, mouse model	Lipid accumulation, adipogenic markers: Active, dose-dependent	Unspecified	[15]	B
Anti-obesity/Metabolic	Amelioration of metabolic disturbance, ovarian dysfunction	Mouse (PCOS model)	Metabolic, hormonal, ovarian markers: Active (dose-dependent)	Unspecified	[20]	B
Antimelanogenic	Decreased melanin content (cAMP/PKA pathway inhibition)	B16F10 melanoma cells	Melanin content, cAMP levels: Active, dose-dependent	Unspecified	[41]	C
Hypoglycemic/Antidiabetic	Hypoglycemic, regulation of carbohydrate metabolism	STZ-induced diabetic rats	Dose-dependent	Unspecified	[80]	B
Hypolipidemic	Hypolipidemic, lower cholesterol/lipids	Rats (diabetic hyperlipidemic)	Dose-dependent	*R*	[81]	B
Insulin secretagogue	Glucose-stimulated insulin secretion (GSIS) via Olfr1259	Mouse β-TC6 pancreatic cells	Concentration-dependent (Ca^2+^, cAMP, insulin)	*R*	[32]	B
Spasmolytic/Smooth muscle relaxation	Spasmolytic activity (tracheal and aortic smooth muscle relaxation)	Guinea pig trachea, rat aorta	Relaxation of carbachol/contractions: Active (dose-dependent)	*S*, *R*	[84]	B
Spasmolytic/Smooth muscle relaxation	Antispasmodic effect (intestinal smooth muscle, voltage-gated Ca^2+^ channel)	Guinea pig ileum	IC_50_ (relaxation of KCl-induced contraction)	*R* > *S*	[77]	B
Spasmolytic/Smooth muscle relaxation	Uterine relaxation (inhibits oxytocin-induced contraction)	Isolated rat uterus + mouse writhing model	Relaxation (IC_50_) + writhing inhibition: Active (dose-dependent)	*R*	[69]	B
Spasmolytic/Smooth muscle relaxation	Tracheal relaxation (As/Hg hypercontraction)	Rat tracheal rings	Relaxation (% of hypercontraction): Active	Unspecified	[86]	B
Spasmolytic/Smooth muscle relaxation	Aortic relaxation (As/Hg hypercontraction)	Rat aortic segments	Relaxation (% of hypercontraction): Active	Unspecified	[85]	B
Spasmolytic/Smooth muscle relaxation	Bovine ruminal smooth muscle contractility modulation	Bovine ruminal strips	Contractility change: Active (dose-dependent)	Unspecified	[83]	B

**Table 11:** Biological activities of carvone: Pesticidal and antiparasitic. Sub-rows represent individual studies. PubChem and ChEMBL assay IDs are cited in the Ref column and are not included in the reference list. Tier: A = comprehensive characterization; B = solid single method; C = screening flag; D = weak/unverified.

Activity Class	Specific Activity	Model System	Quantifier/Value	Enantiomer	Ref	Tier
Antiparasitic	Lethal effects on hydatid cyst protoscoleces	*Echinococcus granulosus protoscoleces*	Scolicidal activity (viability): Active (dose-dependent)	Unspecified	[91]	C
Antiparasitic	Anthelmintic activity	*Haemonchus contortus* (multiresistant)	Egg hatch + larval motility: Active	*R*	[33]	C
Antiparasitic	Anthelmintic	Lambs (*H. contortus* infected)	Fecal egg count reduction: Active	Unspecified	[92]	C
Antiparasitic	Modulation of abamectin anthelmintic activity	Lambs (*H. contortus*, in vivo PK-PD)	Egg count, PK parameters: Synergistic with abamectin	*R*	[78,93]	C
Insecticidal	Contact and fumigant toxicity	*Tribolium castaneum*	LD_50_	*R*	[12]	B
Insecticidal	Repellent activity	*Tribolium castaneum*	% repellency (dose-dependent)	*S*, *R*	ChEMBL ID 103–112	B
Insecticidal	Fumigant insecticidal activity	*Spodoptera littoralis* (cotton leafworm)	LC_50_	*R*	ChEMBL ID 132	B
Insecticidal	Aphidicidal activity	*Rhopalosiphum maidis*, *Sitobion avenae*	IC_50_ (AChE inhibition)	Unspecified	[87]	B
Insecticidal	Contact + fumigant toxicity	*S. oryzae*, *R. dominica*, *T. castaneum*	LD_50_ (contact), LC_50_ (fumigant): Active	*S*, *R*	[13]	B
Insecticidal	Larvicidal activity	Mosquito larvae	LC_50_ (larval mortality): Active	Unspecified	[89]	B
Insecticidal	Enhanced toxicity	*Callosobruchus chinensis*	LD_50_ (contact), LC_50_ (fumigant): Active	Unspecified	[90]	B
Molluscicidal	Contact and fumigant molluscicidal activity	*Theba pisana*	LD_50_ (contact), LC_50_ (fumigant)	*R*	ChEMBL ID 133, 134	B
Nematicidal	Nematicidal activity	*Meloidogyne incognita* J2 larvae	EC_50_ (24 h/48 h/96 h)	*R*	[88]	B

## Data Availability

No new data were created in this study. All data discussed are derived from published literature and public databases (PubMed, PubChem, ChEMBL).

## Supplementary Materials

The following supporting information can be downloaded at: https://www.mdpi.com/article/10.3390/molecules31173106/s1.
