# Supplementary material for: Molecular Targets and Biological Activities of Carvone: An Evidence-Graded Review
Source: Molecules. 2026 Sep 4;31(17):3106. doi: 10.3390/molecules31173106 (PMC13567479; doi:10.3390/molecules31173106)
Supplement: Supplementary file 1 [file molecules-31-03106-s001.zip › molecules-4533083-supplementary.pdf]

PRISMA 2020 Checklist

For: "Molecular Targets and Biological Activities of Carvone: An Evidence-Graded Review"

Manuscript ID: molecules-4533083

Authors: Zdeněk Dvořák, Jiří Hrubý

#	Section	Item	Description and Location in Manuscript
**1**	**Title and Abstract**	Title	*Identify the report as a systematic review.*<br>Reported. The title includes 'An Evidence-Graded Review,' and the Methods section (8.1) explicitly describes the systematic literature search strategy with PRISMA compliance. The abstract follows the PRISMA for Abstracts structure.
**2**		Abstract	*See the PRISMA 2020 for Abstracts checklist.*<br>Reported. The abstract provides the rationale, objective, data sources (PubMed, PubChem, ChEMBL), key findings (45 targets, 99 activities), and conclusions, consistent with the PRISMA 2020 abstract checklist.
**3**	**Introduction**	Rationale	*Describe the rationale for the review in the context of existing knowledge.*<br>Reported. Section 1 (Introduction) describes the rationale: carvone is a pharmacologically significant monoterpene with no prior systematic, evidence-graded catalogue of molecular targets. The existing review by Pina et al. (2022) is cited as the most recent prior effort, with its limitations discussed.
**4**		Objectives	*Provide an explicit statement of the objective(s) or question(s) the review addresses.*<br>Reported. The Introduction states the objective: to systematically compile carvone's molecular targets and biological activities from literature and bioassay databases, assign evidence tiers, and align pharmacological evidence with traditional uses.
**5**	**Methods**	Eligibility criteria	*Specify the inclusion and exclusion criteria for the review and how studies were grouped for the syntheses.*<br>Reported. Section 8.1 specifies: inclusion criteria were original research publications reporting carvone molecular targets or biological activities; exclusion criteria were carvone-derivative studies, duplicates, and non-carvone compounds. The scope was restricted to carvone itself for chemical specificity.
**6**		Information sources	*Specify all databases, registers, websites, organisations, reference lists and other sources searched or consulted to identify studies. Specify the date when each source was last searched or consulted.*<br>Reported. Section 8.1 and 8.3 specify: PubMed (searched through July 2026), PubChem bioassay database (accessed 14 July 2026), ChEMBL activity database (accessed 14 July 2026). Three carvone

			CIDs were queried: 16724 (S-carvone), 439570 (R-carvone), 7439 (racemic).
**7**		Search strategy	*Present the full search strategies for all databases, registers and websites, including any filters and limits used.*<br>Reported. Section 8.1 describes the search strategy: 24 targeted PubMed queries via NCBI E-utilities API (esearch and efetch) combining 'carvone' with target- and activity-specific keywords. Queries were designed to capture both enantiomers and the racemic form. PubChem REST API (pug/assay/aid/AID/CSV) and ChEMBL API (chembl/api/data/activity.json) queries are described in Section 8.3.
**8**		Selection process	*Specify the methods used to decide whether a study met the inclusion criteria, including how many reviewers screened each record and each report retrieved, whether they worked independently, and if applicable, details of automation tools used in the process.*<br>Reported. Section 8.1 describes the selection process: deduplication by PMID, removal of duplicates against existing corpus, exclusion of carvone-derivative studies. The NCBI E-utilities API was used as an automation tool for systematic query execution. Two authors (ZD, JH) reviewed the retained publications for eligibility.
**9**		Data collection process	*Specify the methods used to collect data from reports, including how many reviewers collected data from each report, whether they worked independently, any processes for obtaining or confirming data from study investigators, and if applicable, details of automation tools used in the process.*<br>Reported. Section 8.2 describes the data extraction process: structured tables were used for target and activity entries. Data extraction from PubChem and ChEMBL APIs was performed using BIOMNI, a biomedical AI agent, as an automation tool. Two authors (ZD, JH) manually reviewed and curated all AI-extracted data entries against source publications, as described in the AI-use disclosure in the manuscript back matter. Database records (PubChem AIDs, ChEMBL IDs) were extracted programmatically via API queries.
**10a**		Data items — outcomes	*List and define all outcomes for which data were sought. Specify whether all results that were compatible with each outcome domain in each study were sought, and if not, the methods used to decide which results to collect.*<br>Reported. Section 8.2 defines the data items: target entries capture target name, target identifier (UniProt/Gene), activity type, activity measure and value (Ki, IC50, EC50, Kd, % effect), units, enantiomer, species/model system, assay type, source citation, and confidence grade. Activity entries capture activity class, specific activity, model system, quantifier and value, enantiomer, species, source citation, and confidence grade. All compatible results from each study were sought.
**10b**		Data items — other variables	*List and define all other variables for which data were sought (e.g. participant and intervention characteristics, funding sources). Describe any assumptions made about any missing or unclear information.*<br>Reported. Section 8.2 specifies additional variables: enantiomer identity (S, R, both, or unspecified), assay type, species/model system, and source citation. Missing or unclear information was flagged with the confidence grade (Low) and noted in the tables. Database-only records were tagged with PubChem AID or ChEMBL

			identifier.
**11**		Study risk of bias assessment	*Specify the methods used to assess risk of bias in the included studies, including details of the tool(s) used, how many reviewers assessed each study and whether they worked independently, and if applicable, details of automation tools used in the process.*<br>Reported (adapted). A formal risk-of-bias tool (e.g., Cochrane RoB) was not applied because this review catalogues molecular target interactions and bioactivity profiles rather than clinical intervention outcomes. Instead, a four-tier evidence grading framework (A/B/C/D) was applied to every entry (Section 8.7), incorporating assay type diversity, dose-response characterization, in vivo validation, orthogonal method count, and independent replication as quality dimensions. Two authors (ZD, JH) assigned grades.
**12**		Effect measures	*Specify for each outcome the effect measure(s) (e.g. risk ratio, mean difference) used in the synthesis or presentation of results.*<br>Reported (adapted). Effect measures are potency values: Ki, IC50, EC50, Kd, or percentage effect at specified concentration. These are reported as absolute values with units in the target and activity tables, not as comparative effect estimates, because this review catalogues binding/functional interactions rather than clinical outcomes.
**13a**		Synthesis methods — eligibility for synthesis	*Describe the processes used to decide which studies were eligible for each synthesis (e.g. tabulating the study intervention characteristics and comparing against the planned groups for each synthesis).*<br>Reported. Studies were grouped by molecular target and by biological activity class. All studies reporting a given target or activity were included in the corresponding table section. The evidence-tiering framework (Section 8.7) was applied to stratify entries within each target/activity group.
**13b**		Synthesis methods — data preparation	*Describe any methods required to prepare the data for presentation or synthesis, such as handling of missing summary statistics, or data conversions.*<br>Reported. Section 8.4 describes the qHTS data-quality audit: PubChem assay CSVs were parsed for activity outcome, activity score, phenotype classification, and curve class. ChEMBL activity records were queried for activity comment and assay description. Entries were classified as validated, inconclusive, or artifact. Artifact entries had their curve-fit potency values removed. Unit standardization was applied where necessary.
**13c**		Synthesis methods — tabulation/display	*Describe any methods used to tabulate or visually display results of individual studies and syntheses.*<br>Reported. Results are presented in structured tables (Tables 1–4 for molecular targets; activity tables for biological activities) and a figure showing the distribution of biological activities (Figure 2). Ward's hierarchical clustering was used for visual display of target and activity groupings (Section 8.6, labelled as exploratory).
**13d**		Synthesis methods — synthesis approach	*Describe any methods used to synthesize results and provide a rationale for the choice(s). If meta-analysis was performed, describe the model(s), method(s) to identify the presence and extent of statistical heterogeneity, and software package(s) used.*

			Not applicable. Meta-analysis was not performed because the included studies report heterogeneous assay types, endpoints, and model systems across diverse molecular targets. A narrative synthesis with evidence-tiered tabulation was used instead. Exploratory hierarchical clustering (Ward's method, Euclidean distance, scipy) was performed as a supplementary visualisation, labelled as exploratory and hypothesis-generating (Section 8.6).
**13e**		Synthesis methods — heterogeneity exploration	*Describe any methods used to explore possible causes of heterogeneity among study results (e.g. subgroup analysis, meta-regression).*<br>Not applicable. No meta-analysis was performed; heterogeneity exploration via subgroup analysis or meta-regression is not applicable. The exploratory clustering analysis (Section 8.6) was used to visualise natural groupings but not to formally investigate heterogeneity.
**13f**		Synthesis methods — sensitivity analyses	*Describe any sensitivity analyses conducted to assess robustness of the synthesized results.*<br>Not applicable. No meta-analysis was performed; sensitivity analysis is not applicable. The evidence-tiering framework (Section 8.7) serves as a robustness check by stratifying findings by evidence quality.
**14**		Reporting bias assessment	*Describe any methods used to assess risk of bias due to missing results in a synthesis (arising from reporting biases).*<br>Reported (adapted). Publication bias was addressed by explicitly reporting negative results: NCI-60 inactivity across 55 cancer cell lines and a comprehensive counterscreen of steroid/nuclear receptors, human protein kinases, phosphatases, and HSP90 (all inactive). These negative findings are reported in the manuscript to counter publication bias. No formal statistical test for publication bias (e.g., funnel plot, Egger's test) was applied because no meta-analysis was performed.
**15**		Certainty assessment	*Describe any methods used to assess certainty (or confidence) in the body of evidence for an outcome.*<br>Reported (adapted). The four-tier evidence grading framework (A/B/C/D) described in Section 8.7 serves as the certainty assessment method. Tier A requires multi-assay characterization with in vivo validation; Tier B requires dose-response quantification from peer-reviewed literature; Tier C covers screening-level observations; Tier D covers artifacts and unverified predictions. This framework is analogous to GRADE but adapted for in vitro/in vivo pharmacological target characterization rather than clinical outcomes.
**16a**	**Results**	Study selection — flow	*Describe the results of the search and selection process, from the number of records identified in the search to the number of studies included in the review, ideally using a flow diagram.*<br>Reported. Figure 3 presents the PRISMA flow diagram: 506 unique PubMed publications identified, 80 retained after deduplication and exclusion of carvone-derivative studies. PubChem yielded 1,535 assay records; ChEMBL yielded 136 activity records. Section 8.1 describes the selection process and numbers.
**16b**		Study selection — excluded studies	*Cite studies that might appear to meet the inclusion criteria, but which were excluded, and explain why they were excluded.*<br>Reported. Section 8.1 states that carvone-derivative studies were

			excluded to maintain chemical specificity. The scope restriction to carvone itself (excluding derivatives) is explicitly described.
**17**		Study characteristics	*Cite each included study and present its characteristics.*<br>Reported. All 108 included references are cited in the References section. Study characteristics (target, activity type, potency, model system, enantiomer, assay type) are presented in the molecular target tables (Tables 1–4) and biological activity tables. Each entry includes the source citation number.
**18**		Risk of bias in studies	*Present assessments of risk of bias for each included study.*<br>Reported (adapted). The confidence grade (High, Medium, Low) assigned to each entry in the target and activity tables serves as the study-level quality assessment. The evidence tier (A/B/C/D) assigned to each target provides an aggregate quality assessment across all studies contributing to that target. These are presented in Tables 1–4 and the activity tables.
**19**		Results of individual studies	*For all outcomes, present, for each study: (a) summary statistics for each group (where appropriate) and (b) an effect estimate and its precision (e.g. confidence/credible interval), ideally using structured tables or plots.*<br>Reported (adapted). Results for each study are presented as absolute potency values (Ki, IC50, EC50, Kd) or percentage effects in the target and activity tables. Confidence intervals are not reported because the source studies typically do not provide them for in vitro pharmacological assays. The confidence grade and evidence tier provide qualitative precision indicators.
**20a**		Results of syntheses — summary	*For each synthesis, briefly summarise the characteristics and risk of bias among contributing studies.*<br>Reported. For each molecular target, the tables summarise the number of studies, assay types, potency ranges, and evidence tier. The Discussion section (Section 7) provides narrative summaries of the evidence for key targets (AhR, GABA-A, JNK1, SIRT1, Nrf2, TRPV1, AChE, OR1A1).
**20b**		Results of syntheses — statistical results	*Present results of all statistical syntheses conducted. If meta-analysis was done, present for each the summary estimate and its precision and measures of statistical heterogeneity. If comparing groups, describe the direction of the effect.*<br>Not applicable. No meta-analysis or statistical synthesis was performed. Results are presented as tabulated individual study findings with evidence-tier stratification.
**20c**		Results of syntheses — heterogeneity	*Present results of all investigations of possible causes of heterogeneity among study results.*<br>Not applicable. No formal heterogeneity investigations were conducted (no meta-analysis performed). The exploratory clustering analysis (Section 5) visualised natural groupings but did not formally investigate heterogeneity.
**20d**		Results of syntheses — sensitivity	*Present results of all sensitivity analyses conducted to assess the robustness of the synthesized results.*<br>Not applicable. No sensitivity analyses were conducted (no meta-analysis performed).

**21**		Reporting biases	*Present assessments of risk of bias due to missing results (arising from reporting biases) for each synthesis assessed.*<br>Reported (adapted). Negative results are explicitly reported (NCI-60 inactivity, receptor/kinase counterscreens) to mitigate publication bias. No formal statistical assessment of reporting bias was conducted because no meta-analysis was performed.
**22**		Certainty of evidence	*Present assessments of certainty (or confidence) in the body of evidence for each outcome assessed.*<br>Reported. The evidence tier (A/B/C/D) assigned to each target and activity represents the certainty assessment. Tier A targets (AhR, GABA-A) have the highest certainty; Tier B targets (JNK1, SIRT1, Nrf2, TRPV1, AChE, OR1A1) have solid but incomplete evidence; Tier C and D have lower certainty. These are presented in Tables 1–4 and summarised in Table 4.
**23a**	**Discussion**	Discussion — interpretation	*Provide a general interpretation of the results in the context of other evidence.*<br>Reported. Section 7 (Discussion) interprets the findings in the context of existing carvone literature, comparing the evidence-tiered targets with prior reviews and highlighting the AhR and GABA-A receptor as the most robustly characterized targets.
**23b**		Discussion — limitations of evidence	*Discuss any limitations of the evidence included in the review.*<br>Reported. The Discussion and Conclusions sections discuss limitations: predominance of in vitro data, limited in vivo target-specific validation, reliance on qHTS data with inherent artifact risk, and the absence of clinical trial data for most targets.
**23c**		Discussion — limitations of review processes	*Discuss any limitations of the review processes used.*<br>Reported. The Discussion acknowledges that the review is limited to PubMed-indexed literature (potential for missing non-indexed studies), that the exploratory clustering has weak partition structure, and that PRISMA counts could not be independently re-verified from retrieval logs.
**23d**		Discussion — implications	*Discuss implications of the results for practice, policy, and future research.*<br>Reported. The Conclusions section (Section 9) discusses implications: carvone's AhR antagonism provides a mechanistic basis for traditional topical applications; the evidence tiering identifies priority targets for further investigation; and the framework sets a standard for natural product reviews.
**24a**	**Other information**	Registration and protocol — registration	*Provide registration information for the review, including register name and registration number, or state that the review was not registered.*<br>Not applicable. The review was not registered in a prospective systematic review register (e.g., PROSPERO) because it concerns molecular target characterization and bioactivity profiling of a natural product, not evaluation of a clinical intervention. This is stated in the Methods section (8.1).
**24b**		Registration and protocol — protocol access	*Indicate where the review protocol can be accessed, or state that a protocol was not prepared.*<br>Not applicable. A formal review protocol was not prepared prior to conduct, as the review was an evidence catalogue rather than a hypothesis-testing systematic review. The systematic search strategy

			is fully described in Section 8.1.
**24c**		Registration and protocol — amendments	*Describe and explain any amendments to information provided at registration or in the protocol.*<br>Not applicable. No registration or protocol was prepared; no amendments apply.
**25**		Support	*Describe sources of financial or non-financial support for the review, and the role of the funders or sponsors in the review.*<br>Reported. Funding sources are declared in the Funding section of the manuscript. The authors declare no specific funding for this review.
**26**		Competing interests	*Declare any competing interests of review authors.*<br>Reported. The authors declare no conflicts of interest, as stated in the manuscript.
**27**		Availability of data, code and other materials	*Report which of the following are publicly available and where they can be found: template data collection forms; data extracted from included studies; data used for all analyses; analytic code; any other materials used in the review.*<br>Reported. The data extracted from included studies are presented in full in the manuscript tables. PubChem and ChEMBL data are publicly available via their respective APIs. The PubMed search was conducted via NCBI E-utilities (publicly available). The PRISMA flow diagram (Figure 3) documents the search and selection process. No additional data collection forms or analytic code are required beyond what is described in the Methods.
